# Current composite-feature classification methods do not outperform simple single-genes classifiers in breast cancer prognosis

**DOI:** 10.3389/fgene.2013.00289

**Published:** 2013-12-23

**Authors:** Christine Staiger, Sidney Cadot, Balázs Györffy, Lodewyk F. A. Wessels, Gunnar W. Klau

**Affiliations:** ^1^Life Sciences, Centrum Wiskunde & InformaticaAmsterdam, Netherlands; ^2^Computational Cancer Biology, Division of Molecular Carcinogenesis, Netherlands Cancer InstituteAmsterdam, Netherlands; ^3^Research Laboratory of Pediatrics and Nephrology, Hungarian Academy of SciencesBudapest, Hungary; ^4^Cancer Systems Biology Center, Netherlands Cancer InstituteAmsterdam, Netherlands; ^5^Delft Bioinformatics Lab, Faculty of Electrical Engineering, Mathematics and Computer Science, TU DelftDelft, Netherlands; ^6^Operations Research and Bioinformatics, Faculty of Sciences, VU University AmsterdamAmsterdam, Netherlands

**Keywords:** outcome prediction, breast cancer, classification, feature selection, networks, evaluation

## Abstract

Integrating gene expression data with secondary data such as pathway or protein-protein interaction data has been proposed as a promising approach for improved outcome prediction of cancer patients. Methods employing this approach usually aggregate the expression of genes into new composite features, while the secondary data guide this aggregation. Previous studies were limited to few data sets with a small number of patients. Moreover, each study used different data and evaluation procedures. This makes it difficult to objectively assess the gain in classification performance. Here we introduce the Amsterdam Classification Evaluation Suite (ACES). ACES is a Python package to objectively evaluate classification and feature-selection methods and contains methods for pooling and normalizing Affymetrix microarrays from different studies. It is simple to use and therefore facilitates the comparison of new approaches to best-in-class approaches. In addition to the methods described in our earlier study (Staiger et al., 2012), we have included two prominent prognostic gene signatures specific for breast cancer outcome, one more composite feature selection method and two network-based gene ranking methods. Employing the evaluation pipeline we show that current composite-feature classification methods do not outperform simple single-genes classifiers in predicting outcome in breast cancer. Furthermore, we find that also the stability of features across different data sets is not higher for composite features. Most stunningly, we observe that prediction performances are not affected when extracting features from randomized PPI networks.

## 1. Introduction

During the past decade several algorithms for predicting outcome in breast cancer based on gene expression data were developed. The first predictors used single-genes approaches that extracted genes, which were differentially expressed between the “good” outcome (metastasis-free for at least 5 years) and “poor” outcome patients (metastasis within 5 years). Two prominent gene signatures that were determined by such approaches are the gene signatures by van 't Veer et al. (2002) and Wang et al. (2005). Although these gene signatures can predict outcome, they vary substantially between data sets, and could thus not provide a homogeneous biological interpretation of the data. Moreover, Ein-Dor et al. (2005) showed in their study that there exist many other signatures that perform as well as the suggested gene signatures. This indicates that the signal is distributed over many genes which in turn makes it difficult to pinpoint one predictive network or gene signature from expression data alone. One explanation for this lies in the data. Since the underlying data are high-dimensional gene expression studies that contain many genes but only few patients, the extraction of predictor genes is prone to overtraining and may fit the noise in the data rather than explaining the underlying disease/phenotype.

Integrating gene expression data with secondary data such as pathway or protein-protein interaction (PPI) data has been proposed to address these problems and to improve outcome prediction of cancer patients (Chuang et al., 2007; Lee et al., 2008; Taylor et al., 2009; Abraham et al., 2010; Dao et al., 2010; Ma et al., 2010). These methods infer disease or subtype specific subnetworks and subpathways and use their status as features in classification. In the context of classification we call these subnetworks and subpathways composite features. In the single-genes approaches, each gene is represented by a gene expression vector across the patients, composite features carry a vector in which for each patient the expression values of the feature's member genes are aggregated. Employing composite features reduces the feature space. The underlying biological hypothesis that motivates the data integration and aggregation of genes is that genes do not act alone, and complex diseases, such as cancer, are caused by the activation or inactivation of whole pathways and protein complexes.

**Network Inference**

The article describes a novel framework for evaluating network inference methods in the context of breast cancer. The inferred networks are specific for the outcome of breast cancer patients with respect to the endpoints “5-year distant metastasis free survival” and “5-year recurrence free survival.” We tested the classification performance of classifiers employing the inferred networks as features and compared the performances to classifiers employing single genes. Our results show that the tested classifiers employing network-based features do not perform better than simple single-genes classifiers on the breast cancer data. However, we find evidence that network inference methods are more sensitive to the quality of the underlying data and are thus less noisy.

Previous studies exploring the use of such features were limited to few data sets with a small number of patients. Moreover, each study used different data and evaluation procedures. This makes it difficult to objectively assess the gain in classification performance and shows the need for a standardized evaluation procedure.

To overcome these problems we recently suggested a classification protocol and showed on a breast cancer cohort of ~900 samples that current composite-feature classification methods do not outperform simple single-genes classifiers in predicting outcome in breast cancer (Staiger et al., 2012). Similar findings have been reported in (Cun and Fröhlich, 2012). Furthermore, we showed that the gene signatures defined by composite features are not more stable across different data sets than single genes. We found that, unexpectedly, classifiers employing composite features extracted from randomized PPI networks and pathway databases performed as well as those employing features extracted from unperturbed secondary data. In our evaluation we strictly separated between the training and the testing data by using different gene expression studies for the two steps.

Since the publication of the first composite classifiers, more gene expression data has become available. In addition, procedures to remove batch effects and merge data sets have become available. This allows the creation of much larger breast cancer gene expression data sets, resulting in more statistical power in the analyses. According to the findings by Ein-Dor et al. (2006) thousands of samples are required to generate stable gene lists for classification. In our work we pooled twelve studies to form a data set of 1600 patients. To account for the fact that we now only have one data set, we employ a double loop cross validation (DLCV) protocol (Wessels et al., 2005) that also ensures strict separation between the testing and training data. All classifications are performed by nearest mean classifiers (NMC). We chose the NMC for the following reasons: (i) the NMC provides performances comparable to other classifiers on expression data (Wessels et al., 2005; Popovici et al., 2010), (ii) the NMC is a simple base-line classifier, and (iii) compared to other non-linear classifiers it offers an easier way to biologically interpret the use of features.

In this work, we introduce the Amsterdam Classification Evaluation Suite (ACES), an implementation of the DLCV protocol. ACES is a Python package to objectively evaluate classification and feature-selection methods and contains methods for pooling and normalizing Affymetrix gene expression microarray data from different studies. In the provided software package both schemes, the DLCV and the previously published pipeline (Staiger et al., 2012), can be applied in the evaluation procedure.

ACES is simple to use and therefore facilitates the comparison of new approaches to best-in-class approaches. In addition to the methods described in (Staiger et al., 2012), we include here the well-established prognostic gene signatures proposed by van 't Veer et al. (2002) and Wang et al. (2005), the recent composite-feature selection method by Dao et al. (2010) and two network-based gene-ranking methods by Morrison et al. (2005) and by Winter et al. (2012). To analyse classification performances we employ a much larger cohort of patients. In contrast to the paired data set evaluation in Staiger et al. (2012) we describe here an evaluation framework that makes use of a DLCV, which facilitates the evaluation of classifiers on one large data set. Furthermore, we provide a concise correction for batch effects. In addition to the above-mentioned NMC, the software package contains an implementation of the logistic regression and the *k*-nearest neighbor classifier. To account for new developments in the field we provide detailed information on how to add new data to the package. Furthermore, we dedicate a tutorial on how to insert new feature-selection methods into ACES.

Applying ACES to a large breast cancer cohort confirms the findings of our previous study, that is, (i) none of the evaluated methods performs better than a simple single-genes classifier; (ii) features extracted by the methods are as stable as single genes, and (iii) randomizing the secondary data source has no effect on the classification performance.

The software package ACES, the normalization and merging package for gene expression data and all raw results can be downloaded from http://ccb.nki.nl/aces/.

## 2. Materials and methods

### 2.1. Classification

**Figure 1:**
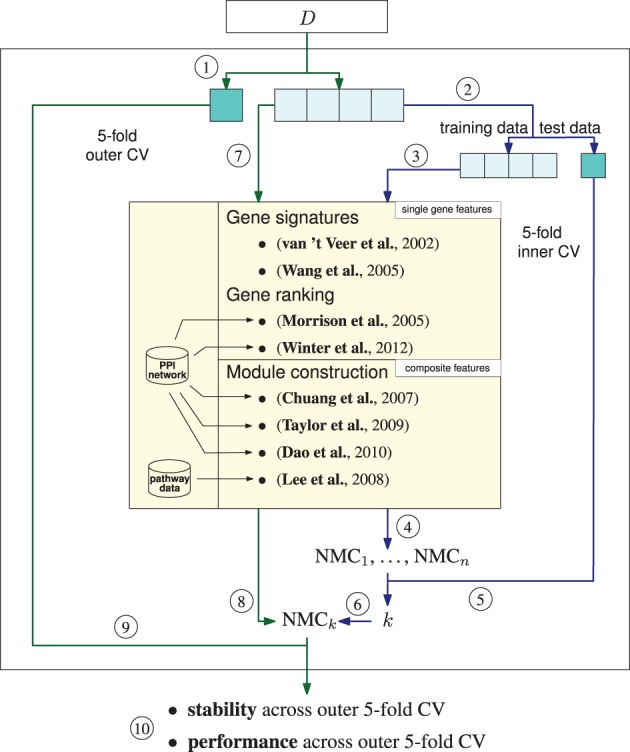
**Amsterdam classification evaluation suite (ACES)**. ACES executes a double-loop cross validation (DLCV) to train classifiers with features extracted by different feature extraction methods. The DLCV consists of two nested fivefold cross-validations (CV), an outer and an inner CV. **1**, Data set *D* is split into five parts of which one is reserved as test data and four parts are used as the training data for the outer CV loop. To determine the best number of features an inner CV is executed. The blue arrows denote the connection between the outer and the inner cross validation. Inner CV (blue arrows): **2**, The training data of the outer CV is split into five parts, four parts serve as training data, the remaining part is used as test data for the inner CV. **3**, Features are determined with one of the methods listed in the yellow box. The methods returns *n* ranked features—either single genes or composite features. Note that due to significance testing the number of returned features is not known in advance for some of the methods. We set the maximum for *n* to 400. **4**, Nearest mean classifiers (NMC) are trained by sequentially adding the features according to their ranking. The index corresponds to the number of features employed in the training. Thus, NMC*_i_* is an NMC trained on the top *i* features. **5**, The performance of the NMCs is tested on the reserved test data of the inner CV. **6**, Steps 3 and 4 are repeated until each of the five splits was used as test data yielding five performances for each number of features. The index *k* of the best performing NMC gives the number of features that will be employed in the outer CV. Outer CV (green arrows): **7**, *k* features are extracted on the four training splits, i.e., the training data of the outer CV. **8**, An NMC with the top *k* ranked features is trained on the training data. **9**, The classifier's performance is tested on the fifth split. **10**, After completing the outer CV, i.e., each split was employed once as test data, we receive five performances and five sets of features.

Classifiers were trained by a double-loop cross validation (see Figure 1). Since the gene signatures (Wang et al., 2005) and (van 't Veer et al., 2002) consist of a fixed set of genes, it was not necessary to run the inner CV. Hence, only one classifier for each training data set was trained employing all genes in the gene signatures. All other feature selection methods provide a ranking of the features. We trained classifiers with increasing number of features up to 400 features. Features were added sequentially to the classifiers according to the order in the ranking.

The package provides the nearest mean classifier (NMC) with four different scoring metrics based on the cosine distance and the Euclidean distance. Here we use a metric (V1), that projects the sample to the straight line connecting the two class means and normalizes the value; points that project closer to the mean of the poor outcome patients *μ*_poor_ are scored as zero, points that project closer to the mean of the good outcome μ_good_ patients are scored as one. The three other metrics are described in Supplement section 8. We also provide the code for a *k*-nearest neighbor classifier and the logistic regression.

### 2.2. Expression data

We compiled a large cohort of breast cancer samples from NCBI's Gene Expression Omnibus (GEO) (see Table 1) as it was suggested in (Györffy and Schäfer, 2009). We only took samples from the U133A platform into account and removed duplicate samples, that is, samples that occur in several studies under the same GEO id. Array quality checks were executed for all samples belonging to the same study by the R package arrayQualityMetrics. Due to high memory demands of this package, studies containing more than 400 samples had to be divided into two parts. Samples that were classified as outliers in the RLE or NUSE analysis were discarded. Finally, all samples across all studies were normalized together using R's justRMA function yielding for each sample and each probe a log(intensity) value. This normalization also included a quantile normalization step. Subsequently, probe intensities were mean centered, yielding for each sample and each probe *p* a $\log(\frac{\text{intensity}}{\mu ({\text{intensity}}_{p})})$ value.

**Table 1:** **Datasets**.

**Label**	**Data set**	**Geo accession (GSE)**	**No. of poor**	**No. of good**
DMFS	Ivshina	4922	6	29
	Hatzis-Pusztai	25066	102	48
	Desmedt-June07	7390	36	146
	Miller	3494	7	33
	Schmidt	11121	24	145
	Loi	6532	15	32
	Total		190	433
RFS	Ivshina	4922	30	72
	Hatzis-Pusztai	25066	102	48
	Desmedt-June07	7390	56	127
	Minn	2603	21	44
	Miller	3494	21	68
	WangY-ErasmusMC	2034	88	169
	Schmidt	11121	24	145
	Pawitan	1456	33	114
	Symmans	17705	37	187
	Loi	6532	24	33
	Zhang	12093	9	112
	WangY	5327	10	42
	Total		455	1161

*Shown are the original studies from which the two data sets U133A-DMFS and U133A-RFS were compiled. The patient labels “good” and “poor” correspond to 5 year distant metastasis free survival (DMFS) and recurrence free survival (RFS)*.

We found batch effects within single studies, where samples have been collected from different locations and batch effects between studies. Specifically for breast cancer, samples also form batches according to the five subtypes of breast cancer: luminal A, luminal B, Her2 enriched, normal like and basal like. To account for these effects we employed R's combat, where the cancer subtype was modeled as an additional covariate to maintain the variance associated with the subtypes. To do so we needed to stratify the patients according to the subtype. Since this variable is not always available in the annotation of the patients, we predict the subtype employing the PAM50 marker genes as documented in R's genefu package.

Principal component analysis of the batch corrected data revealed pairs of samples with a very high correlation (>0.9). Those pairs were regarded as replicate samples. For each pair of replicate samples one sample was removed randomly. Affymetrix probe IDs were mapped to Entrez Gene IDs via the mapping files provided by Affymetrix. Only probes that mapped to exactly one Gene ID were taken into account and probes starting with AFFX were discarded. If an Entrez Gene ID mapped to several Affymetrix probe IDs, probes were considered in the following order according to their suffix (Gohlmann and Talloen, 2010): “_at,” “s_at,” “x_at,” “i_at,” and “a_at.” When there were still several probes valid for one Gene ID, the Affymetrix probe with the higher variance of expression values was chosen.

The patients' class labels corresponding to recurrence free or distant metastasis free survival were calculated with respect to a 5-year threshold. The final cohort is shown in Table 1. We derived two data sets: one labeled according to recurrence free survival (RFS) and one labeled according to distant metastasis free survival (DMFS). Note, that the DMFS data set is a subset of the RFS data set.

We provide all of the code, data, secondary data and the procedure for normalization, sample selection and batch correction as a package at http://ccb.nki.nl/aces/.

### 2.3. Secondary data

#### 2.3.1. KEGG

We collected all pathway information for *Homo sapiens* (hsa) from the KEGG database (Kanehisa et al., 2010), version December 2010. The considered pathways are metabolic pathways, pathways involved in genetic information processing, signal transduction in environmental information processing, cellular processes and pathways active in human disease and drug development. We obtained 215 pathways. In this way we obtained a network composed of 200 pathways containing 4066 nodes and 29972 interactions of which 3249 nodes are also contained in the expression sets.

#### 2.3.2. MsigDB

As second pathway database we used the C2 collection of the MsigDB (Subramanian et al., 2005) (version 3.0), which was also used in Lee et al. (version 1.0). It contains gene sets from pathway databases such as KEGG, gene sets made available in scientific publications and expert knowledge. We obtained 3272 gene sets of which 3000 could be entirely or partially covered by genes in the expression data. The MsigDB does not contain any edges, thus this database was only usable for the algorithm by Lee et al. (2008).

#### 2.3.3. HPRD9

The protein-protein interactions were derived from the literature. We employed the HPRD version 9 (Prasad et al., 2009). The HPRD contains 9231 proteins and 35853 interactions. The protein ids were mapped to their corresponding Entrez Gene IDs. There are 7728 genes contained in both the HPRD and the expression sets.

#### 2.3.4. OPHID/I2D

The OPHID/I2D database, downloaded in April 2011, combines protein-protein interactions from BIND, HPRD and MINT as well as predicted interactions from yeast, mouse and *C. elegans*. The database contains 12643 nodes and 142309 edges. 10018 of the nodes are also present in the breast cancer studies examined here.

#### 2.3.5. PPI network curated by Chuang et al. (NetC)

Chuang et al. (2007) gathered a PPI of 57228 interactions and 11203 nodes of which 8572 are contained in the cohort. The source of the interactions are yeast two hybrid experiments and interactions predicted from co-citation.

### 2.4. Feature selection methods

Let 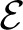 be the expression data matrix where 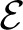
*_pj_* is the expression of gene *j* in patient *p*. The set of genes is denoted by *G*. We denote the patient's class label by *c_p_* where *c_p_* = 0 indicates a “good” outcome patient and *c_p_* = 1 indicates a “poor” outcome patient. Similarly, we denote the patient's survival time as *t_p_* ∈ ℝ.

A PPI network is defined as a graph 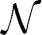 = (*G, E*) where *G* is the set of genes and edges *E* denote interactions between genes. A pathway is an unsorted set of genes *G*′ ⊆ *G*.

#### 2.4.1. Gene signatures Wang et al., 2005 and van 't Veer et al., 2002

We included two gene signatures for predicting distant metastasis free survival based on gene expression data, the signature by van 't Veer et al. (2002) and by Wang et al. (2005). Each gene *j* is used as one feature in the classifier and the value for each of these features is the gene's expression value for a patient *p*. Both signatures are actually probe signatures. The signature by Wang et al. (2005) (Erasmus) was determined on the Affymetrix U133A array, thus all probes are also present in the two data sets we generated. The 76 probes map to only 66 unique geneIDs.

The signature by van 't Veer et al. (2002) (NKI) was determined on an Agilent platform. This required the probes to be matched to gene IDs and then mapped to the data. Here we employed the gene ID collection from the MsigDB ‘VANTVEER_BREAST_CANCER_POOR_PROGNOSIS’ pathway as gene signature. From this pathway 41 genes were also present in the two data sets.

#### 2.4.2. Single-genes and random genes—the benchmark methods

The single-genes approach ranks all genes *G* by their *t*-statistic between the good and poor outcome patients. The top *n* genes are used in an NMC and the expression values of the top *n* genes serve as the feature values for each patient. To determine the genes to be employed in a random single-genes classifier we simply randomly selected *n* genes from the total set of genes.

#### 2.4.3. GeneRank (Morrison et al., 2005) and Winter et al., 2012

The GeneRank algorithm (Morrison et al., 2005) and the method by Winter et al. (2012) are based on Google's page rank algorithm (Page et al., 1999). The vector of gene ranks *r* is calculated as follows:

(1) $$(I-dW^{t}D^{-1})r=(1-d)r^{0}$$

where *I* is the identity matrix, *W^t^* is the transpose of the PPI network's adjacency matrix, *D* = diag(deg(*j*) + 1) for *j* ∈ G and *r*^0^ is the vector of initial ranks. The vector *r* contains for each gene the resulting rank. The degree of genes was incremented by one to allow singleton genes to be included in the calculation. The parameter *d* is called the damping factor and regulates the influence of the network on the rank. If *d* = 1 gene ranks are determined by the network only whereas with *d* = 0 each gene keeps its initial rank.

As initial ranks for GeneRank we chose the absolute difference of average expression between the “poor” outcome patients and the “good” outcome patients, as it was suggested in the original paper. Additionally, we calculated classification performances with the initial ranks being the *t*-statistic between the two patient groups.

The original Winter method proposed the correlation coefficient between the survival times of the patients and the genes' expression values. Additionally, we considered the correlation between the patients' class labels.

#### 2.4.4. Chuang et al., 2007

This method determines subnetworks with the aim to distinguish between “good” and “poor” outcome patients. The discriminatory power of a subnetwork is evaluated by the mutual information score between the discretized average gene expression (Equation 2) and the patients' class labels. Given a subnetwork induced by *G*′ ⊆ *G*, its activity score *a* for a patient *p* is given by

(2) $$a_{G^{\prime },p}=\sum_{j\;\in \;G^{\prime }}\frac{e_{pj}}{\sqrt{\left|G^{\prime }\right|}}$$

To calculate the mutual information of a subnetwork we need to calculate the activity scores for each patient and subsequently discretize them. Let *a*′ be the vector of discretized activity scores for the network induced by *G*′ and let *c* be the vector of class labels. The mutual information score for the subnetwork is defined as

(3) $$s_{\text{MI}}(a^{\prime },c)=\sum_{x\;\in \;a^{\prime }}\sum_{y\;\in \;c}\rho (x,y)\log\frac{\rho (x,y)}{\rho (x)\rho (y)}$$

where ρ denotes the joint and marginal probability density functions.

All subnetworks are subjected to statistical tests assessing the significance with respect to the local and global null distribution of the activity scores and with respect to the null distribution of mutual information scores. We used the java package PinnacleZ as an implementation of the algorithm. PinnacleZ performs a z-normalization prior to the subnetwork search, which is depreciated in a fivefold cross validation. Therefore, we implemented a patch that skips this normalization step.

#### 2.4.5. Taylor et al., 2009

This algorithm identifies differentially coordinated hub proteins in the PPI network. As measure for coordination the Pearson correlation is used. The coordination of a hub and one of its interactors is defined as the Pearson correlation *PC*(*h, i*) between the hub's expression *h* and the interactor's expression *i*. To assess the different coordination of a hub across the two patient groups the average hub difference is calculated

(4) $$d(h)=\frac{\sum_{i\;\in \;n(h)}\left|PC^{0}(h,i)-PC^{1}(h,i\right)|}{\left|n(h)\right|}$$

given the two sample classes, indicated by the superscript 0 and 1, *n*(*h*) denotes the set of neighbors. All hubs are subjected to a statistical test, testing the significance of the hub difference. Only hubs with a significant hub difference are selected as features. Feature values for each patient are given by the average difference of expression between the hub and its interactors.

#### 2.4.6. Dao et al., 2010

This method defines subnetworks that obey two criteria: they are (i) maximally densely connected and (ii) show deregulation in at least *L* poor outcome patients. To decide whether a gene is deregulated the expression matrix is discretized, i.e., each pair of patient and gene is assigned one of the three signs {+, −, 0}, where + means the gene is overexpressed, − indicates underexpression and 0 indicates that patient does not show an aberrant gene expression with respect to the cohort. Given a PPI network and a gene expression data set the algorithm first enumerates all connected subnetworks that obey the above-mentioned two criteria such that no subnetwork is a subgraph of any other subnetwork. The subnetworks are ranked based on their information gain. The parameter *L* was set such that at least 5% of the poor outcome patients were covered by each subnetwork. In the classification step these subnetworks served as features. To classify patients the average expression across all member genes of each subnetwork was calculated for each patient to obtain feature values.

#### 2.4.7. Lee et al., 2008

This method extracts sub-pathways as features from a pathway database. The member genes of each pathway are ranked by their *t*-statistic between the “good” and “poor” outcome patients. Then the top *n* genes are combined by Equation 2 and their combined expression is again tested by the *t*-statistics. The search for the subpathway starts with the highest ranking gene and successively adds the next genes in the ranking as long as the *t*-statistic increases.

## 3. Tutorials

To enable a wider use of ACES and to keep the package flexible to new developments in the field we provide tutorials on how to include more expression data, PPI networks and pathway data. Further, we dedicate one tutorial to the topic of including more feature extraction methods, including methods that are developed in programming languages different from Python, and show how to create a wrapper that links the new software to ACES.

### 3.1. Integrating new data

We created Python objects to represent the expression data, PPI networks and pathways. The class ExpressionDataset contains the expression matrix, patient labels and the patient class labels. PPI networks are represented by the class EdgeSet. Each edge is represented by a frozenset containing the start and end node of the edge. Weights on the edges can be stored as a dictionary in EdgeSet.edgeweights, where the key is the edge and the value is the weight. Pathways are represented by the class GeneSetCollection. The whole pathway database is represented as a list of lists, GeneSetCollection.geneSets, where each pathway itself is stored as a list of genes. The names of the pathways are stored as a list in GeneSetCollection.geneSetsNames.

#### 3.1.1. Expression data

The Python script NewDatasets.py provides code and information on how to convert external data files into an ExpressionDataset and subsequently saves it in hdf5 format.

#### 3.1.2. Network and pathway data

New PPI data should be provided as SIF formatted file and can be read in by EdgeSet.ReadSIF. Similarly pathway data can be read in by GeneSetCollection. ReadGeneSetCollection. The file format is as follows. Each line contains one gene set, and genes in a gene set are space-separated. If you want to attach names to each gene set, insert a line starting with “NAME” directly before the gene set. Examples are provided in the folder “experiments/data” in the ACES package.

### 3.2. Integrating a new feature selection method

We assume that any new feature selection method written in some programming language is provided as software that is called from command line. We further assume that all input is read in from files and all output is written to files.

To integrate a new feature selection method you will need to provide the code for the two classes Feature ExtractionFactory and FeatureExtractor. The FeatureExtractorFactory determines the features on a training data set and a secondary data source, whereas the FeatureExtractor maps the input genes from the data set to the feature space and scores each feature for each sample in the data set. We clearly divided between these two classes since they correspond to different steps in the pipeline.

#### 3.2.1. The FeatureExtractorFactory

In the FeatureExtractorFactory the code that defines features is provided. When the actual feature extraction algorithm is given as an independent software package in a different language the FeatureExtractionFactory serves as a wrapper to connect the software to ACES. To initialize a new FeatureExtractorFactory the location of the executable of the software is passed to the constructor—the __init__ function:

def __init__(self, softwareExecutable):
     self.executable = softwareExecutable



The method train receives all necessary data instances to extract the features. To ensure that several instances of the FeatureExtractionFactory can be run at the same time on the same machine we first create a temporary directory to which the input files are written. The input files can be directly created from the data instances, which contain functions to write the data as space- or tab-separated files. The format for pathways is as follows: each line contains all genes belonging to one pathway separated by spaces. The name of each pathway, if present in the GeneSetCollection instance, is printed in the line preceding the member genes and is indicated by the keyword “NAME.” Instances of the type EdgeSet can be written to a space-separated sif-file or a file where each line consists of the start node, end node and the edge weight. The function ExpressionDataset.writeToFile writes the gene expression matrix to a tab-separated file, while all patients' class labels are saved in a separate file by the function ExpressionDataset.writeClasslabels.

In the example below the expression matrix is written to the file “matrix_file.txt,” the patients' class labels are written to “classlabels_file.txt” and the network is written to “network_file.sif”:

def train(self, dataset, network):
    tempdir = tempfile.mkdtemp()

    MatrixFilename = os.path.join(tempdir,
                  'matrix_file.txt')
    dataset.writeToFile(MatrixFilename)
    ClassesFilename = os.path.join(tempdir,
                  'classlabels_file.txt')
    dataset.writeClasslabels(ClassesFilename)
    NetworkFilename = os.path.join(tempdir,
                  'network_file.sif')
    network.writeSIF(NetworkFilename)



Next, we create the command that calls the executable with the input files. Note that the executable lies in a different directory than the input files. To achieve that also the output is written to the temporary directory we either need to copy the executable to the new location or create an option for the output in the executable. The shutil module provides several functions for copying files to a different location from within python. For now, we assume the executable is located in the temporary directory and the output is written to a file called “output.txt” that contains the features. The list args contains the complete call of the executable. You can check the correctness by print ' '.join(args). The command is executed as subprocess in the temporary directory:

def train(self, dataset, network):

    tempdir = tempfile.mkdtemp()

    …

    args = []
    args.extend([yourCompiler+' '+os.path.basename
        (executable)])
    args.extend([MatrixFilename, ClassesFilename,
        NetworkFilename])

    proc = subprocess.Popen (args, cwd=os.path.
      dirname(tempdir))



Finally, the generated output.txt needs to be read in and formatted as a list of lists, where each sublist contains the genes belonging to one feature. This is accomplished by modules = readOutput(tempdir+'/output.txt'), which must be provided by the user. In ACES we assume that the genes belonging to the features are not given by their name but by their index with respect to the data set used in the function train. Thus, if genes are given by name in the output file, we need to map them to their indices:

def train(self, dataset, network):

    …

    modules = readOutput
              (tempdir+'/output.txt')
    geneLabelsToIndex = dict(zip(dataset,
                        geneLabels, xrange(len(dataset.
                        geneLabels))))
    features = [frozenset([geneLabels
               ToIndex[gene] for gene in module if
               gene in geneLabelsToIndex]) for module
               in modules]

        return NewFeatureExtractor
        (dataset.geneLabels, features)



The output of the FeatureExtractorFactory is an instance of the FeatureExtractor that maps an expression data set with the same genes and the same ordering of the genes as the data set employed in the train function to the feature space.

#### 3.2.2. The FeatureExtractor

In the FeatureExtractor an input data set is mapped to the feature space and each feature is scored for each patient of the data set. Features are defined over the indices of the genes in the data set employed to determine the features. The FeatureExtractor is initialized with the gene space and the features it maps to. Only data sets with the same genes and the same ordering of genes can be mapped to the features:

def __init__(self, geneLabels, features):

        self.geneLabels         = geneLabels
        self.features           = features
        self.validFeatureCounts = range(1,
                   len(self.features) + 1)



The method extract maps the data to the first *k* features. We ensure here that there are *k* features and that the data set is defined on the correct genes:

def extract(self, dataset, k):

	assert all(dataset.geneLabels ==
    self.geneLabels)
	assert k in self.validFeatureCounts

	return numpy.transpose(numpy.array ([self.score
             (dataset.expressionData, feature)
             for feature in self.features[:k]]))



The function score attaches a score to each feature for each patient. In the case of single genes this would be the gene's expression value for the patients. In case of a feature consisting of multiple genes the function score needs to provide information on how to merge the genes' expression to one value. We show here an example of how to average over the genes' expression that belong to the same feature:

@staticmethod
def score(expressionData, feature):
    return numpy.sum(expressionData[:, list(feature)],
    axis = 1) /len(feature)



To store and reload a feature extractor efficiently, we provide a function toJsonExpression which stores all the information in a json document:

def toJsonExpression(self):
    return json.dumps((self.__class__.__name__,
	[geneLabel for geneLabel in self.geneLabels],
	[sorted(feature) for feature in self.features]))



The full example code is shown in Supplementary section 6.

## 4. Results and discussion

### 4.1. Network and pathway-based methods do not outperform the benchmark methods

We evaluated the performances of nearest mean classifiers (NMC) employing the benchmark feature-selection methods “single genes,” “random genes” and gene signatures specific for breast cancer outcome, “NKI” and “Erasmus,” and compared them with the performances of classifiers employing composite features.

All classifiers were trained in the double-loop cross validation (DLCV) procedure described in Figure 1. The DLCV consists of two nested fivefold cross validations. In the outer CV we determine the training and testing data. From the inner CV we obtain the parameters for the outer CV's classifier and feature selection method (number of features and the damping factor for the Page Rank based algorithms Morrison et al., 2005; Winter et al., 2012). Once the inner CV is completed we use its best performing parameters to train the outer CV classifier. Thus, although having only one initial data set for training and evaluating classifiers, we strictly separate the data employed in these two steps, which ensures an unbiased evaluation.

**Figure 2:**
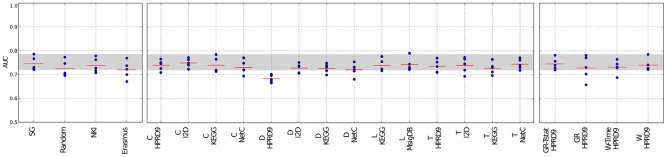
**Classification results of the NMC V1 on the DMFS data set**. The number of features was trained in the inner CV. Shown are the five performances received in the outer CV. The individual AUC values are shown as blue dots. The red lines indicate the mean performances. The gray area indicates the performance interval of the single-genes classifier. SG, Single genes; L, Lee; C, Chuang; T, Taylor; D, Dao; W, Winter using association between class labels and gene expression as initial gene ranks; W-time, Winter using association between survival times and gene expression as initial gene ranks; GR, GeneRank with absolute expression difference between “good” and “poor” outcome groups as initial rank; GR-Tstat, GeneRank employing the *t*-statistic as initial gene ranks.

**Figure 3:**
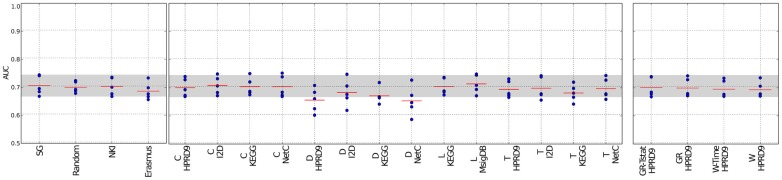
**Classification results of the NMC V1 on the RFS data set**. Description as in Figure 2. SG, Single genes; L, Lee; C, Chuang; T, Taylor; D, Dao; W, Winter using association between class labels and gene expression as initial gene ranks; W-time, Winter using association between survival times and gene expression as initial gene ranks; GR, GeneRank with absolute expression difference between “good” and “poor” outcome groups as initial rank; GR-Tstat, GeneRank employing the *t*-statistic as initial gene ranks.

Figures 2, 3 and Supplementary figure S1 show the results for the NMC using the V1 metric. There are no differences in performances between the different versions of the NMC. From this we conclude that the distance measure does not play a major role (the raw data for all NMCs can be downloaded at http://ccb.nki.nl/aces/). None of the composite-features classifiers significantly outperforms the single-genes classifier (see Table S1). In the Supplement sections 2.1–2.19 we show that changing the number of features does not lead to a change in performance. The feature selection proposed by Winter et al. (2012) and the GeneRank algorithm are also influenced by the damping factor. Supplementary section 3, however, shows that the classifiers performances do not vary significantly across different damping factors. This suggests that the network only has a marginal influence on the classification result.

The method by Dao et al. (2010) performs worse than the benchmark methods. The reason might be that not necessarily all patients are considered during extraction of predictive network markers. In the algorithm a minimum number of “poor” outcome patients is required to be covered by each network. However, there is no constraint reinforcing that each patient is covered by the networks. This allows that the same group of poor outcome patients determines all the features and good outcome patients are neglected in this step. Thus, valuable information about patients might be lost, which, in turn, leads to higher misclassification rates.

Previously, we have shown that classifiers employing the features by Taylor et al. (2009) perform worse than the single-genes classifiers (Staiger et al., 2012). In our earlier interpretation of the algorithm each edge was regarded as a single feature. This led to an enormous feature space and to poor classification performances. Here, we keep the selection of hubs and their interactors, but in contrast to the previous classifier, we score each hub by the average expression difference between itself and all of its interactors. This decreases the feature space and leads to much better classification results. Still, the method does not outperform the benchmark methods.

### 4.2. Network and pathway-based methods do not produce more stable gene sets than the benchmark methods

In addition to the claim that using composite features increases classification performance, it is often stated that these features are by far more stable than single genes. Here, we analyze the overlap of composite features by means of Fisher's exact test and compare them to the overlap of single genes. Since composite features consist of many genes we considered all genes belonging to the *k* best performing features. Thus, the overlap of two composite-feature sets is determined by the overlap of the corresponding gene sets. Composite features are calculated from PPI and pathway data, which contain different numbers of genes and fewer genes than there are genes in the expression data. These differences have to be taken into account when comparing the overlap between gene sets. For example, when determining two composite feature sets from the KEGG database for two different data sets the overlap between the two sets is very likely to be higher than generating two feature sets for the same data on the I2D network due to the difference in size of the two PPIs. Fisher's exact test takes these differences into account. We illustrate the use of the test in Supplementary section 7. Moreover, to compare the overlap of the composite features' gene sets to single genes we have to correct for the size of the composite features since a single composite feature can contain many genes. For each training data set and each feature selection approach we obtain the *n* highest ranking features containing *m* genes. We then determine the size-matched highest scoring single genes on the same training data set of the outer CV.

**Figure 4:**
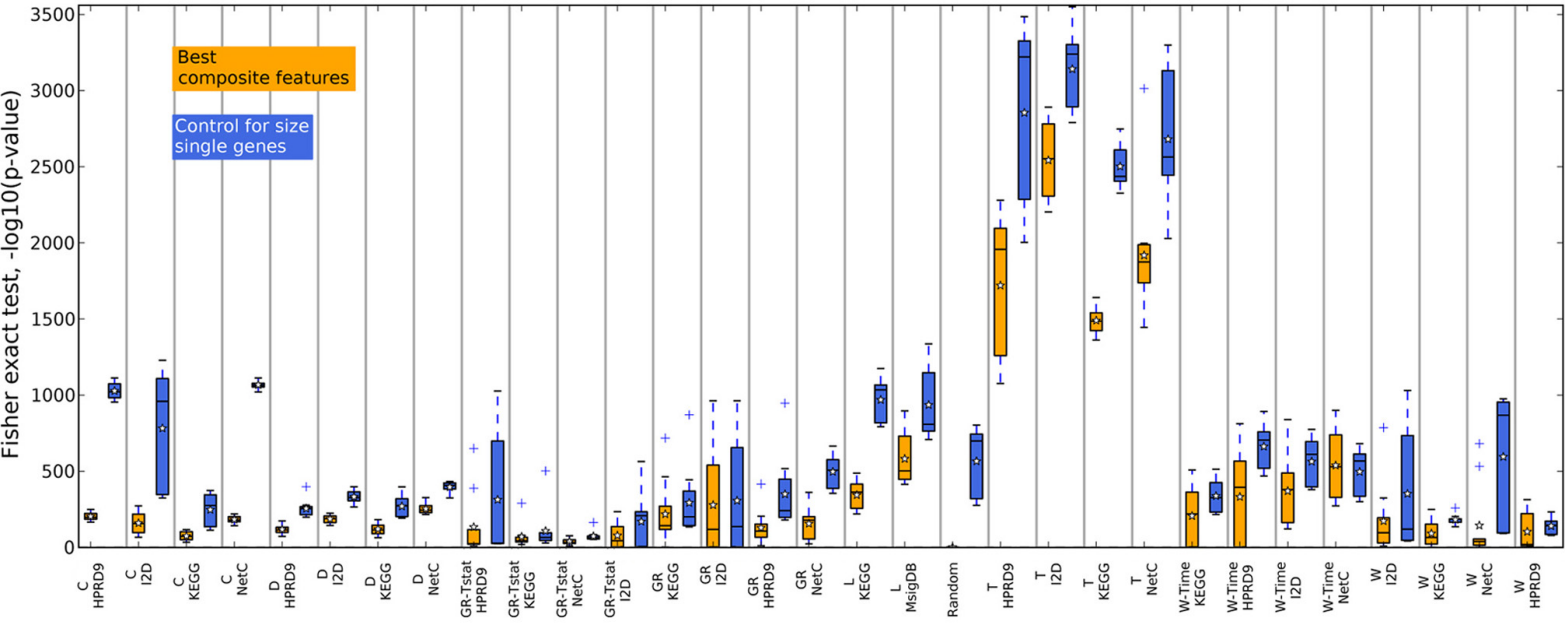
**Overlap of the gene sets corresponding to the best number of features on the DMFS data set**. Genes belonging to the best number of composite features were extracted for each training set (outer CV). The overlap between the gene sets was calculated with Fisher's exact test. The corresponding number of top ranking single genes for each training data set were drawn and the overlap was calculated between these gene sets to control the influence of the gene set sizes.

**Figure 5:**
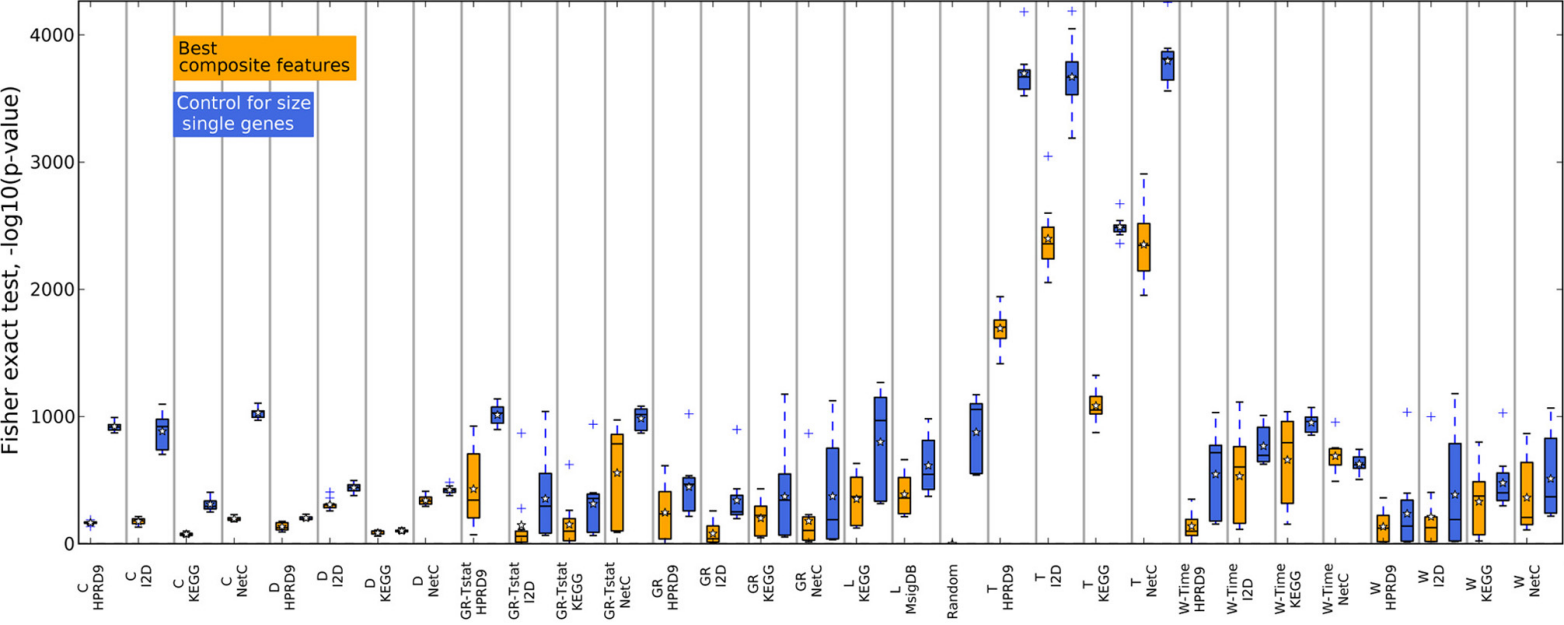
**Overlap of the gene sets corresponding to the best number of features on the RFS data set**.

Figures 4, 5 show that none of the composite features produces more stable gene sets than the single genes. In many cases the control-for-size single genes are more stable than the corresponding composite features. The overlap of randomly drawn genes is very low, as expected. Although their performance in classification is equally good as single genes, the experiment clearly shows that overlap and performance in classification are not related to each other. The method by Taylor et al. (2009) produces highly stable gene sets. The method selects hub proteins and a feature consists of the hub and all of its interactors. Thus, a large number of genes contribute to a feature. This seems to be enough to ensure a high overlap as the corresponding box of the control-for-size single genes also indicate highly stable gene sets.

From the results we can not conclude that composite-features ensure more stable gene signatures from expression data than single-genes classifiers where the genes were selected on an individual basis, given that a sufficiently large number of genes are selected.

### 4.3. Randomization of the secondary data sources does not decrease classification performances of network and pathway-based methods

To find out whether the quality of the PPI networks have a major influence on the performance we executed randomization experiments. The nodes in each network were shuffled. By this the network topology stayed the same, but nodes that were originally hubs may now have only few neighbors and nodes with few neighbors might become hubs. For each PPI network we did this random shuffling 25 times, resulting in 25 PPI networks. Each network dependent method was then executed on each of these 25 networks using the DLCV ACES protocol. Thus, the network provides only non-sensical biological information, which in turn should hinder the methods to extract useful features. We would expect that the classification performances drop dramatically when employing these features.

**Figure 6:**
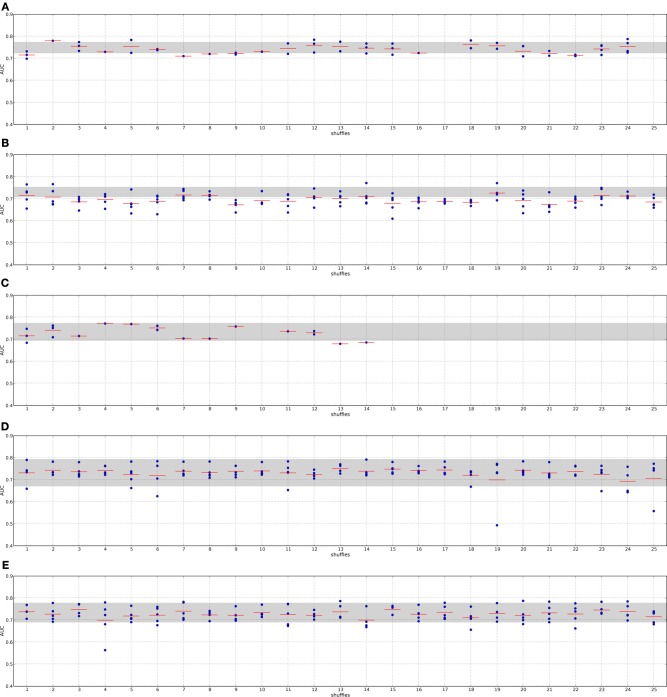
**Classification performance when employing randomized networks, I2D and DMFS data**. The nodes in the I2D PPI network were shuffled 25 times yielding 25 different randomized networks. Each network dependent feature selection method was applied to each of the randomized networks and classifiers were trained using the double-loop CV protocol. The gray area indicates the AUC value interval employing the original PPI network. Panel **(A)**, Chuang; panel **(B)**, Dao; panel **(C)**, Taylor; panel **(D)**, GeneRank with absolute expression difference between “good” and “poor” outcome groups as initial rank; panel **(E)**, Winter using association between survival times and gene expression as initial gene ranks.

**Figure 7:**
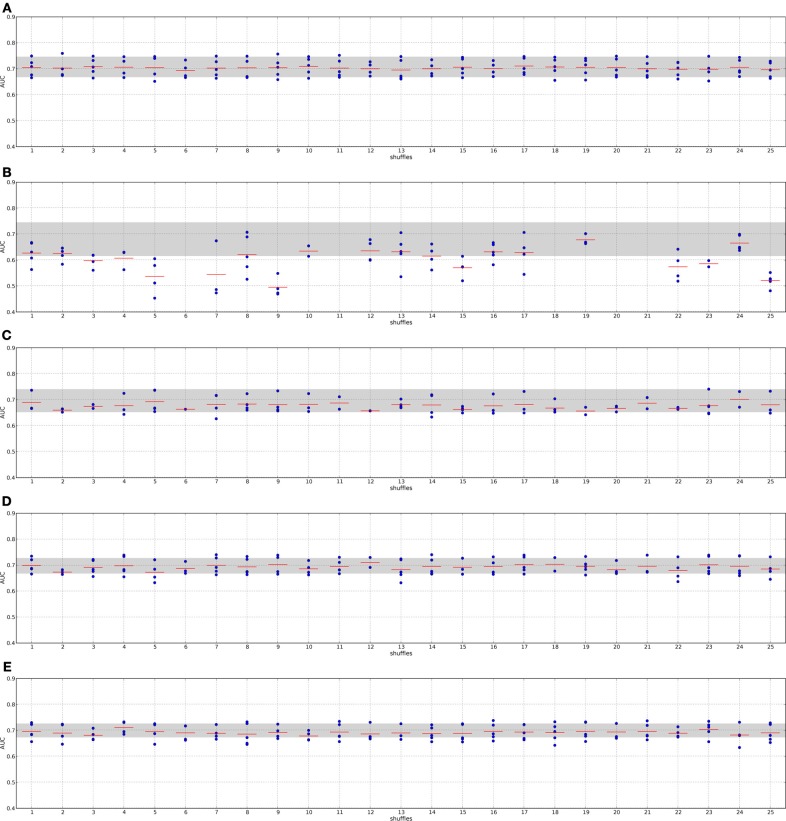
**Classification performance when employing randomized networks, I2D and RFS data**. Description as in Figure 6. Panel **(A)**, Chuang; panel **(B)**, Dao; panel **(C)**, Taylor; panel **(D)**, GeneRank with absolute expression difference between “good” and “poor” outcome groups as initial rank; panel **(E)**, Winter using association between survival times and gene expression as initial gene ranks.

Figures 6, 7 show the results for the network dependent methods executed on the shuffled I2D PPI network, the performance interval employing the original networks is depicted in gray. Figures S2, S3 show the results for the randomized HPRD9.

The methods by Chuang et al. (2007), Dao et al. (2010) and Taylor et al. (2009) do not always find features for some combinations of data set and randomized networks, i.e., the algorithms do not return features. This indicates that these methods are indeed sensitive to the quality of the network data.

The method by Taylor et al. (2009) searches for significantly altered hubs across the two conditions. Shuffling the nodes in the networks disrupts the connection between significantly altered genes and hubs. Previous hub genes might no longer be hubs or may be shifted to a neighborhood in which their interactors do not show high (anti-)correlation with it. Under these circumstances the method cannot define features. A confirmation of this effect provides the analysis of the features. Supplement section 5 clearly shows that the algorithm finds fewer features with fewer member genes on the randomized PPI networks. The effect becomes stronger when a small network is randomized (cf. Taylor on I2D and HPRD9) or when the data set size is small (cf. Taylor on the DMFS data set and the RFS data set). Thus, searching for altered hubs might offer a good biological interpretation of the data in context of outcome prediction. However, it is important to note that the algorithm is sensitive to the network size and data set size. When features can be defined by the method, they perform as well in classification as features determined on the real PPI networks. Thus, the major factor contributing to a good classification performance is the expression data.

Chuang et al. (2007) determines subnetworks whose mutual information between the member genes' expression and the class labels is high. This link is certainly disrupted by randomizing the PPI network. The algorithm includes statistical tests to only return significantly altered subnetworks, which should prevent returning randomized features. Thus, for some combinations of randomized networks and expression data no subnetworks can be found whose mutual information score is significantly high. However, if features are found we observe that the classification performance is as good as with features extracted from the real networks. Moreover, Supplement section 5 shows that the number of features increases on the randomized PPI networks. One reason for that could be that many genes are involved in breast cancer and many of them also show a significant differential expression (Ein-Dor et al., 2005). Thus, by shuffling the nodes there is still a high chance that subsets of these genes again form a subnetwork that is then identified by the algorithm as a feature. Since genes are no longer grouped according to their pathway, the information is scattered over the network. Thus, features extracted from randomized networks with the method by Chuang et al. may contain a lot of redundant information. As above this leads to the conclusion that the main factor in classification is the expression data.

In contrast to the above mentioned algorithms, the features by Dao et al. (2010) perform significantly worse in classification when they were determined on random PPI networks. We also observe that no features are found for some combinations of input data and in general fewer features are found (Supplement section 5). Since it is required in the method that a certain percentage of “poor” outcome patients show deregulation for each of the features, the number of member genes in the features can not decrease. The method searches for maximally densely connected subnetworks that cover at least 5% of the poor outcome patients. As noted before, looking for features that only describe one condition and do not consider information about all training samples might lead to a poor performance. The effect is worsened when giving the algorithm non-sensical biological information, as we do with the randomized networks. However, comparing the results obtained on the I2D network and the HPRD9 network and on the two different expression data sets, it seems that this effect is also linked to network and data set size. Since the methods by Taylor et al. (2009) and Dao et al. (2010) are more sensitive to the underlying quality of the data we can conclude that they are less prone to extract noise from the underlying data.

Also the GeneRank algorithm (Morrison et al., 2005) and the method by Winter et al. (2012) do not suffer from randomizing the networks. Both methods determine the rank for each gene by an initial rank and the diffused ranks of the genes in the vicinity. Having many differentially expressed genes in a network may contribute to selecting genes that can well distinguish between the patient classes. This is also confirmed by the fact that the damping factor, and thus the network, has only a minor influence on the classification when employing real PPI networks (see Section 3 in the supplement).

### 4.4. Composite features extracted from randomized networks are less stable

**Figure 8:**
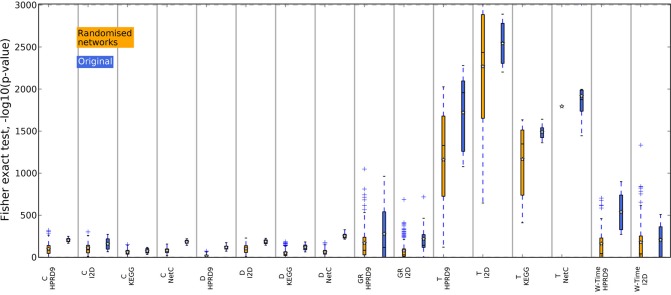
**Overlap of the gene sets determined on the randomized PPI networks, DMFS data set**. The overlap between the gene sets was calculated with Fisher's exact test. The blue boxes show the overlap of the corresponding features determined on the original networks.

**Figure 9:**
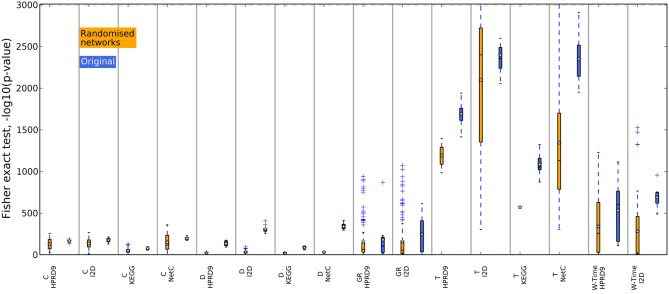
**Overlap of the gene sets determined on the randomized PPI networks, RFS data set**. The overlap between the gene sets was calculated with Fisher's exact test. The blue boxes show the overlap of the corresponding features determined on the original networks.

In previous studies the overlap between features, i.e., in case of composite features the genes contained in the features, has been used as an indicator for biological meaningful features. When genes are chosen as features or as a part of composite features on different data sets, they might contain valuable biological information. We now analyze the overlap between features generated on the randomized PPI networks. For each training data set in the outer CV we determined the best performing features on one randomized network. We then calculated the overlap between the genes contained in the features for the five training data sets as above. Thus, we only compared features that were generated using the same algorithm and the same randomization of the network. The boxes in Figures 8, 9 summarize all values across the 25 randomizations. Overlap for gene sets determined on random networks is always significantly worse than the overlap of features determined on the real networks when employing the method by Dao et al. (2010). Apparently, looking for maximally densely connected subnetworks is an adequate mathematical translation to define marker genes for breast cancer outcome. Taylor always produces an equally stable overlap. The only exception on the NetC PPI network is due to the small number of features that could be determined on this network. This confirms that the high overlap is merely due to the algorithm. Selecting many genes leads to stable gene sets. The results for Chuang, Winter and the GeneRank algorithm are mixed. Here, the stability of features seems to depend on the combination of network and expression dataset. To conclude, we showed that randomizing the subnetworks leads to a loss of information that is important to extract gene sets that are stable across different data sets. However, the lost information is irrelevant for the classification as shown in the previous section.

### 4.5. Summary

Previously many feature selection methods were put forward for better classification of breast cancer outcome. The novel methods claimed that integrating gene expression data and secondary data, such as PPI networks and pathway data, improves the classification performance and provides more stable features. We evaluated the methods based on two large breast cancer data sets and a variety of PPI networks and pathway databases. Our results do not confirm any of these claims.

To facilitate an easy and unbiased evaluation of more methods on more networks, pathways and expression data, we have proposed the Amsterdam Classification Evaluation Suite (ACES), a novel evaluation framework. In the implemented pipeline, we strictly separate between the training data and the testing data by employing a double-loop cross validation procedure. We provide tutorials which make it very easy to extend the described pipeline with additional data. Furthermore, we provide a tutorial and in depth instructions how to include new feature selection methods. ACES is freely available.

We conclude that it remains difficult to evaluate whether the composite-features selection methods draw any useful information from the secondary data sources, such as PPI networks and pathway data. We showed here and in our previous work (Staiger et al., 2012) that the methods by Chuang et al. (2007), Winter et al. (2012) and Lee et al. (2008) and the GeneRank algorithm (Morrison et al., 2005) do indeed perform as well on randomized PPI networks as on the real PPI networks. In contrast, the methods by Dao et al. (2010) and Taylor et al. (2009) are more dependent on the subnetwork structure when selecting features and fail to provide useful features on randomized network data. However, we also observe that in some cases these two methods perform worse on the original PPI networks than the single-genes classifiers, suggesting that some specific combinations of gene expression data and network data delivers less information for the classification task than the expression data alone. This suggests that the most predictive power for outcome is derived from the gene expression data and that the PPI network and pathway data only provides some means to reduce the feature space but adds little to the predictive accuracy of the classifiers. To this end it is extremely difficult to decide whether networks in general add little information to the classification task or whether the tested methods are not able to successfully leverage this information.

There are two independent goals when creating feature selection methods for outcome prediction in breast cancer: (i) to correctly classify the patients and (ii) to find genes or combinations of genes that carry some biological meaning. We have shown that currently the first goal can best be achieved by applying simple single-gene approaches and not by applying elaborate methods that use network or pathway data. However, for the definition of gene signatures specific for certain phenotypes, such methods seem to be more reliable to extract less noisy features—and thus possibly biological meaningful genes—than single-gene approaches.

## Author contributions

Christine Staiger, Lodewyk F. A. Wessels, and Gunnar W. Klau conceived and designed the experiments. Christine Staiger and Balázs Györffy acquired and analyzed the data. Christine Staiger and Sidney Cadot performed the experiments. Christine Staiger, Lodewyk F. A. Wessels, and Gunnar W. Klau analyzed the experimental results. Christine Staiger, Lodewyk F. A. Wessels, and Gunnar W. Klau wrote the manuscript. All authors read and approved the final version of the manuscript.

### Conflict of interest statement

The authors declare that the research was conducted in the absence of any commercial or financial relationships that could be construed as a potential conflict of interest.
