# Supplementary material for: Current composite-feature classification methods do not outperform simple single-genes classifiers in breast cancer prognosis
Source: Front Genet. 2013 Dec 23;4:289. doi: 10.3389/fgene.2013.00289 (PMC3870302; doi:10.3389/fgene.2013.00289)
Supplement: Supplementary file 1 [file DataSheet1.PDF]

Supplement - Current composite-feature classification methods do not outperform simple single-genes classifiers in breast cancer prognosis

Christine Staiger[1,2], Sidney Cadot[2], Balázs Győrffy[3], Lodewyk FA Wessels[2,4,5,*] and Gunnar W Klau[1,6,*]

[1]Life Sciences, Centrum Wiskunde & Informatica, Amsterdam, The Netherlands
[2]Bioinformatics and Statistics, The Netherlands Cancer Institute, Amsterdam, The Netherlands
[3]Research Laboratory of Pediatrics and Nephrology, Hungarian Academy of Sciences, Budapest, Bókay u.53, H-1083, Hungary
[4]Cancer Systems Biology Center, The Netherlands Cancer Institute, Amsterdam, The Netherlands
[5]Delft Bioinformatics Lab, Faculty of Electrical Engineering, Mathematics and Computer Science, TU Delft, Delft, The Netherlands
[6]VU University Amsterdam, Amsterdam, The Netherlands
[*]Corresponding author

November 18, 2013

1 Network and pathway-based networks perform equally well as the benchmark methods

1.1 GeneRank and Winter on KEGG, NetC and I2D

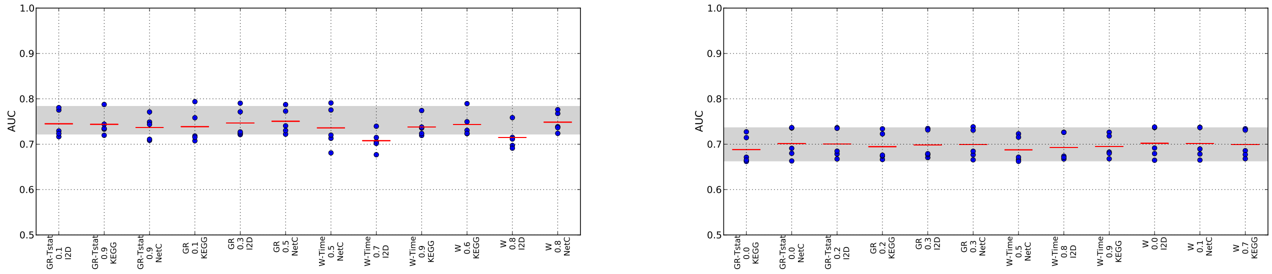

Figure 1: Classification results for the Winter and GeneRank methods on the PPI networks KEGG, HPRD and NetC

1.2 Paired Wilcoxon rank test

To evaluate if any composite classifier performs significantly different from the Single genes classifier we determined the p-values of the paired Wilcoxon rank test. Table S1 shows the raw values, i.e. the values are not corrected for multiple testing. After Bonferroni correction (p-value $*33$) none of the values stays significant.

Table 1: **P-values of the paired Wilcoxon rank test** We compared the AUC value distribution of the Single genes NMC V1 with the values of all composite features NMC V1.

Data set	Method	p-value	Data set	Method	p-value
RFS			DMFS		
	C_HPRD9	0.1250		C_HPRD9	0.6250
	C_I2D	0.8125		C_I2D	0.4375
	C_KEGG	0.8125		C_KEGG	0.0625
	C_NetC	0.6250		C_NetC	0.0625
	D_HPRD9	0.0625		D_HPRD9	0.0625
	D_I2D	0.1250		D_I2D	0.1250
	D_KEGG	0.1250		D_KEGG	0.1875
	D_NetC	0.1250		D_NetC	0.3125
	Erasmus	0.1250		Erasmus	0.1250
	GR-Tstat_HPRD9	0.1250		GR-Tstat_HPRD9	0.8551
	GR-Tstat_I2D	0.1875		GR-Tstat_I2D	0.6250
	GR-Tstat_KEGG	0.3125		GR-Tstat_KEGG	0.4375
	GR-Tstat_NetC	1.0000		GR-Tstat_NetC	0.4375
	GR_HPRD9	0.1875		GR_HPRD9	0.4375
	GR_I2D	0.3125		GR_I2D	0.6250
	GR_KEGG	0.1875		GR_KEGG	0.6250
	GR_NetC	0.6250		GR_NetC	0.4375
	L_KEGG	0.4375		L_KEGG	0.0625
	L_MsigDB	0.0625		L_MsigDB	1.0000
	NKI	0.1875		NKI	0.3125
	Random	0.4375		Random	0.0625
	T_HPRD9	0.0625		T_HPRD9	0.1041
	T_I2D	0.3125		T_I2D	0.4375
	T_KEGG	0.0625		T_KEGG	0.1250
	T_NetC	0.1875		T_NetC	0.8125
	W-Time_HPRD9	0.1250		W-Time_HPRD9	0.3125
	W-Time_I2D	0.3125		W-Time_I2D	0.1250
	W-Time_KEGG	0.1250		W-Time_KEGG	0.1875
	W-Time_NetC	0.1250		W-Time_NetC	0.4375
	W_HPRD9	0.1250		W_HPRD9	0.6250
	W_I2D	0.1875		W_I2D	0.6250
	W_KEGG	0.3613		W_KEGG	1.0000
	W_NetC	0.1875		W_NetC	0.0625

2 Number of features does not have a major influence on the performance

In subsection 2.1 to 2.18 we show the full AUC versus number of features curves for the outer CV loop results. The plots show that the number of features has only minor influence on the actual performance measured in AUC. The GeneRank algorithm and the method by Winter et al. are depending on two parameters the number of features and the damping factor. We found that neither had a major influence on the performance. For these methods we only show the AUC versus number of features curves for the best damping factor if the damping factor is unequal to 0. The figures containing the AUC versus number of features curves for all other damping factors are available upon request.

2.1 Lee

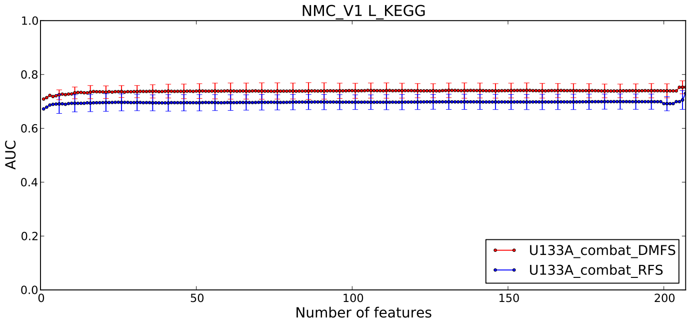
NMC_V1 L_KEGG
AUC
Number of features
U133A_combat_DMFS
U133A_combat_RFS

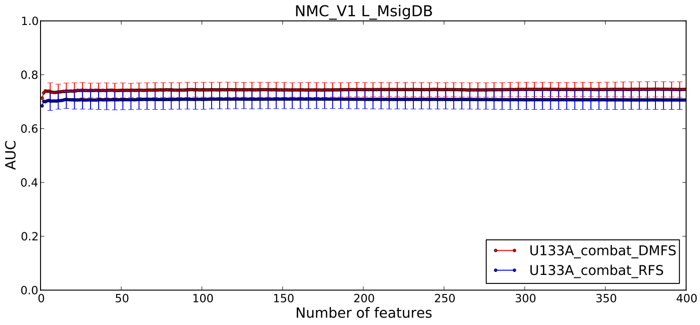
NMC_V1 L_MsigDB
AUC
Number of features
U133A_combat_DMFS
U133A_combat_RFS

2.2 Chuang

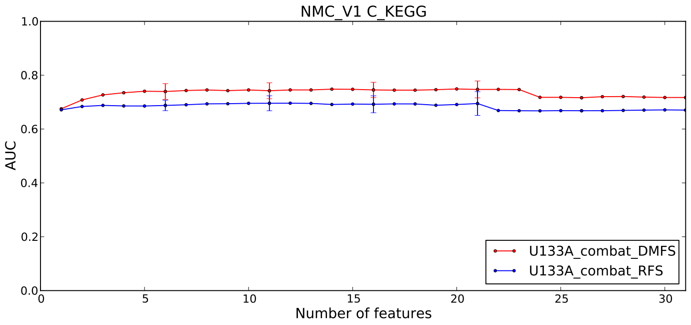
NMC_V1 C_KEGG
AUC
Number of features
U133A_combat_DMFS
U133A_combat_RFS

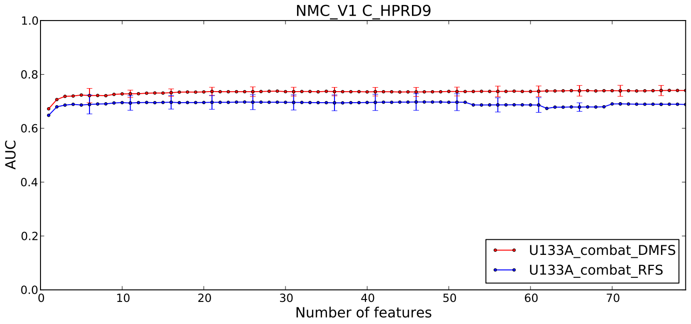
NMC_V1 C_HPRD9
AUC
Number of features
U133A_combat_DMFS
U133A_combat_RFS

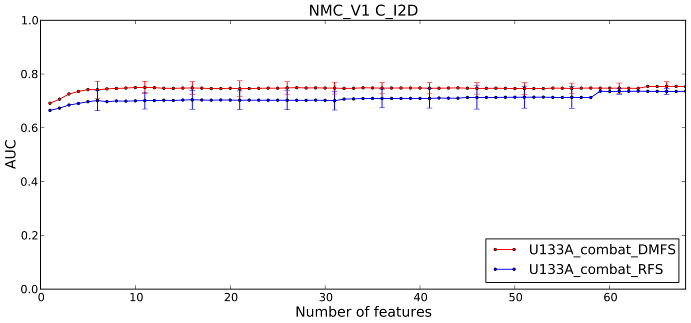
NMC_V1 C_I2D
AUC
Number of features
U133A_combat_DMFS
U133A_combat_RFS

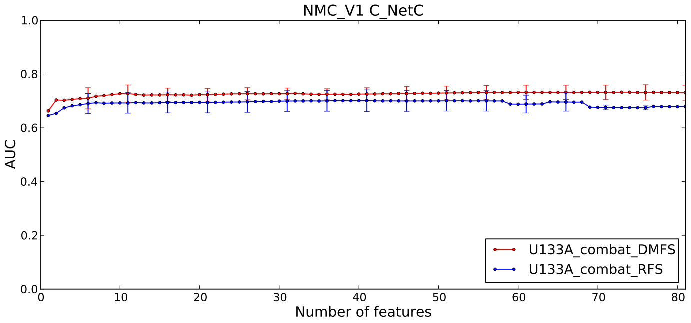
NMC_V1 C_NetC
AUC
Number of features
U133A_combat_DMFS
U133A_combat_RFS

2.3 Dao

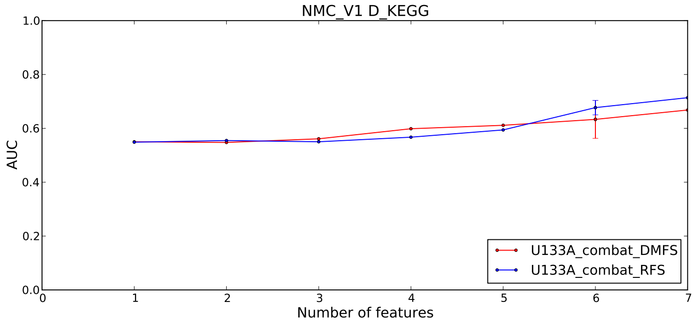

NMC_V1 D_KEGG
AUC
Number of features
U133A_combat_DMFS
U133A_combat_RFS

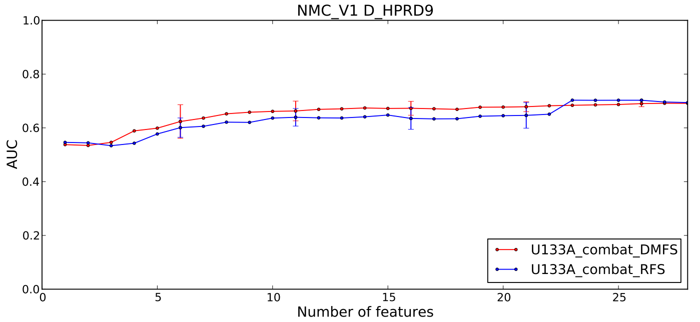

NMC_V1 D_HPRD9
AUC
Number of features
U133A_combat_DMFS
U133A_combat_RFS

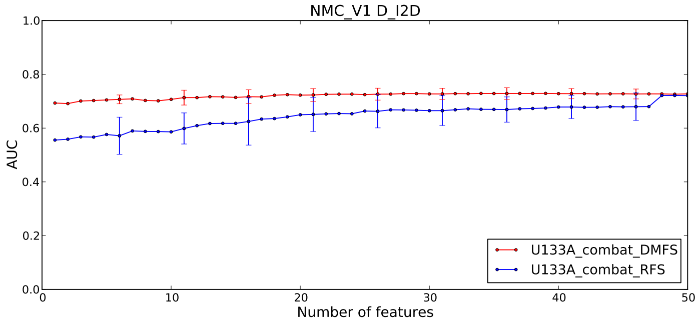

NMC_V1 D_I2D
AUC
Number of features
U133A_combat_DMFS
U133A_combat_RFS

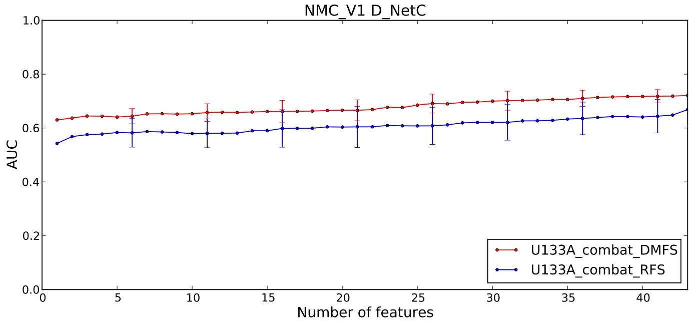

NMC_V1 D_NetC
AUC
Number of features
U133A_combat_DMFS
U133A_combat_RFS

2.4 Taylor

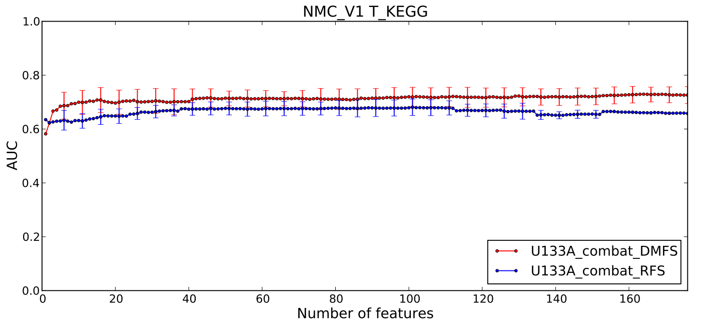
NMC_V1 T_KEGG
AUC
Number of features
U133A_combat_DMFS
U133A_combat_RFS

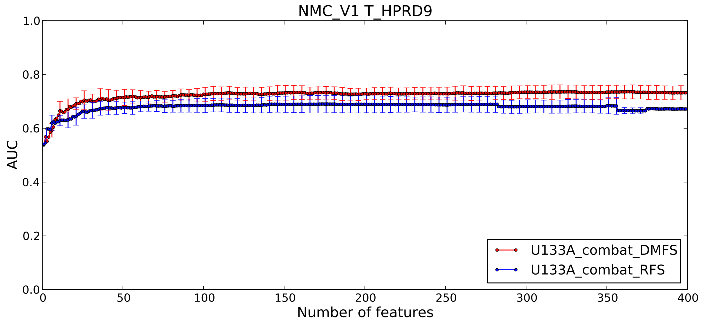
NMC_V1 T_HPRD9
AUC
Number of features
U133A_combat_DMFS
U133A_combat_RFS

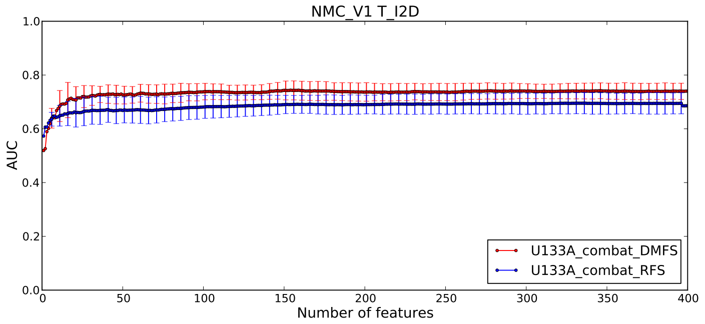
NMC_V1 T_I2D
AUC
Number of features
U133A_combat_DMFS
U133A_combat_RFS

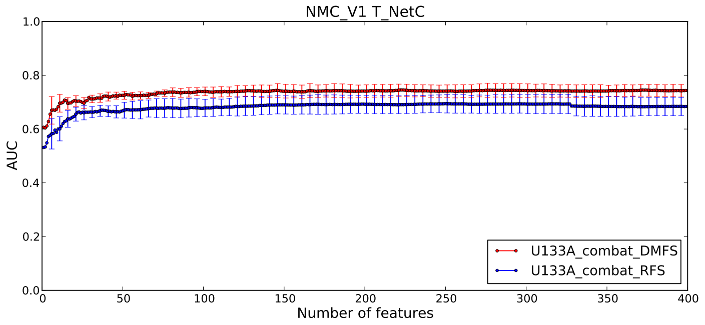
NMC_V1 T_NetC
AUC
Number of features
U133A_combat_DMFS
U133A_combat_RFS

2.5 GeneRank-Tstat - KEGG and NetC, damping factor 0.9

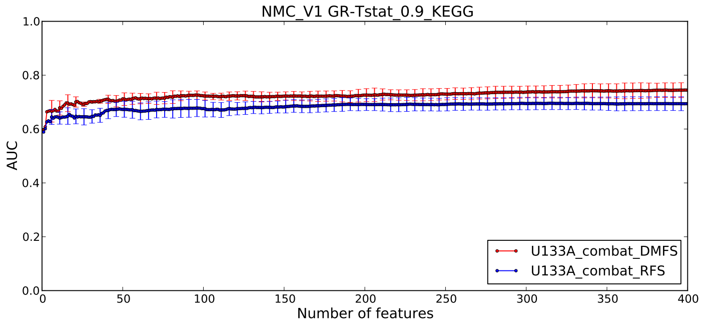

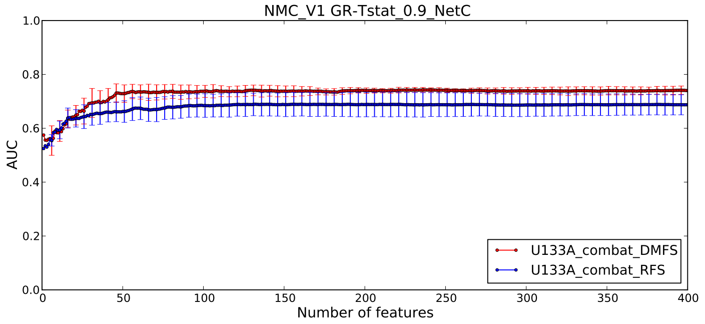

2.6 GeneRank-Tstat - I2D, damping factors 0.1 and 0.2

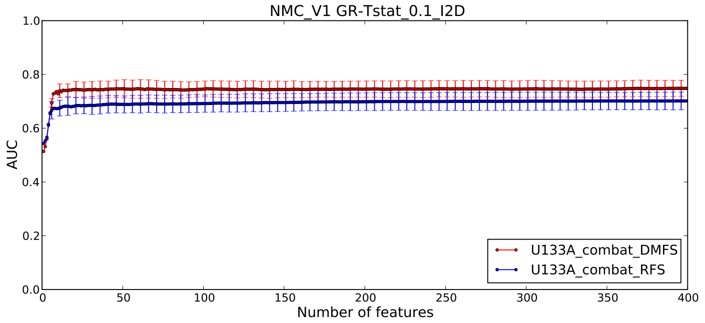

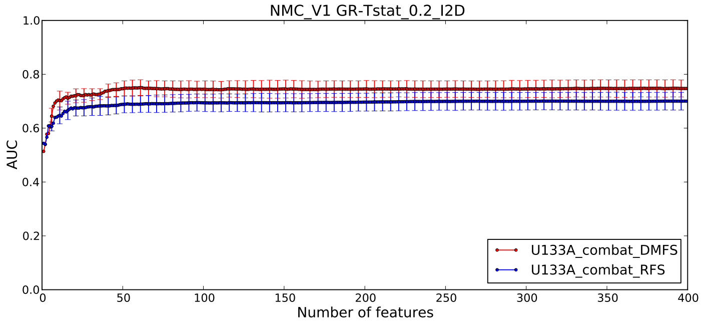

2.7 GeneRank-Tstat - HPRD9, damping factors 0.2 and 0.6

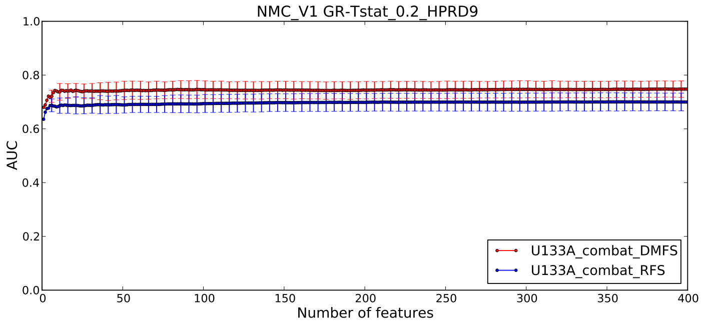

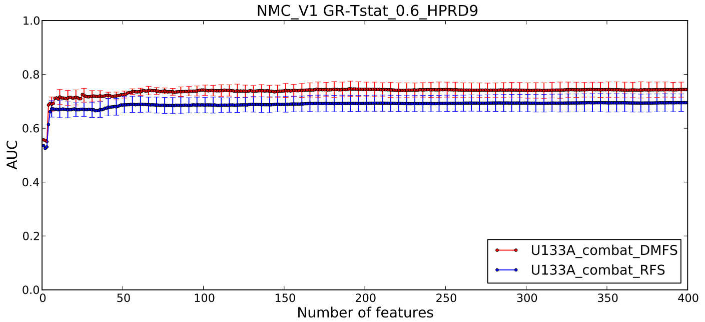

2.8 GeneRank - KEGG, damping factors 0.1 and 0.2

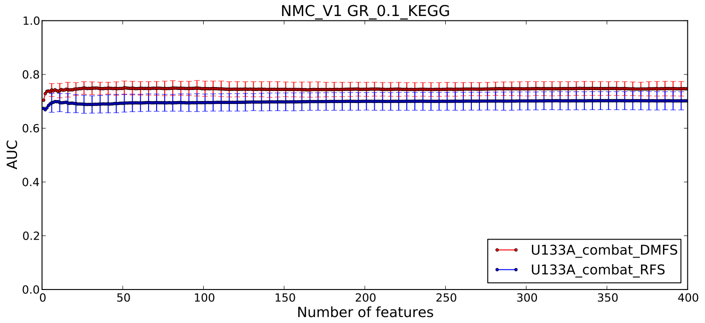

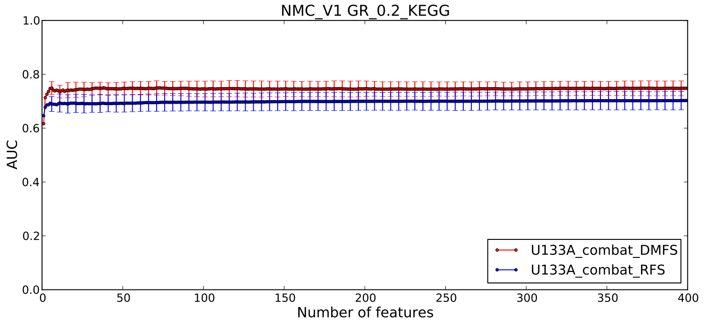

2.9 GeneRank - NetC, damping factors 0.3 and 0.5

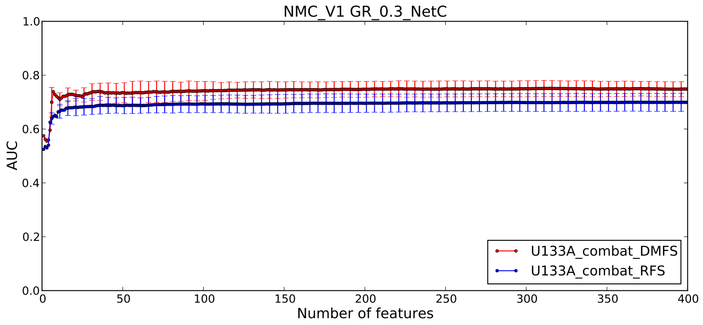

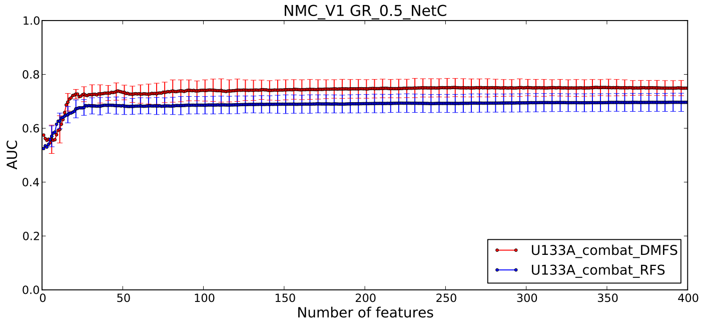

2.10 GeneRank - HPRD9, 0.1 and 0.2

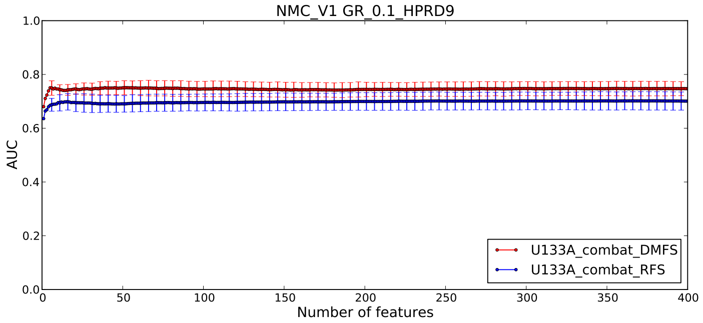

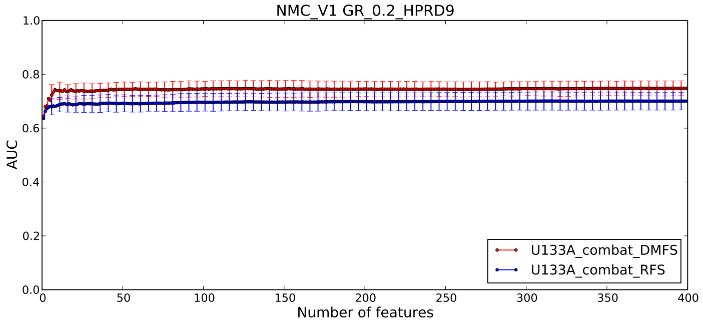

2.11 GeneRank - I2D, damping factor 0.3

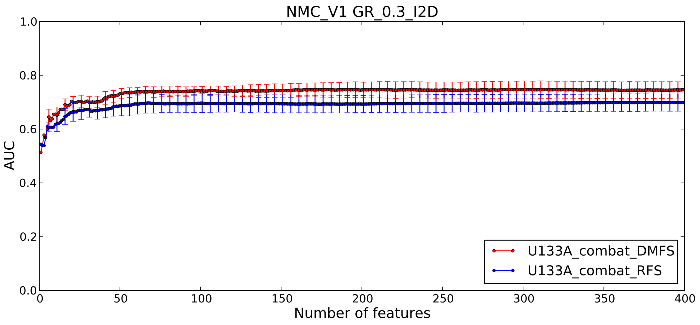

2.12 Winter-Time - KEGG, damping factor 0.9; Winter-Time - NetC, damping factor 0.5

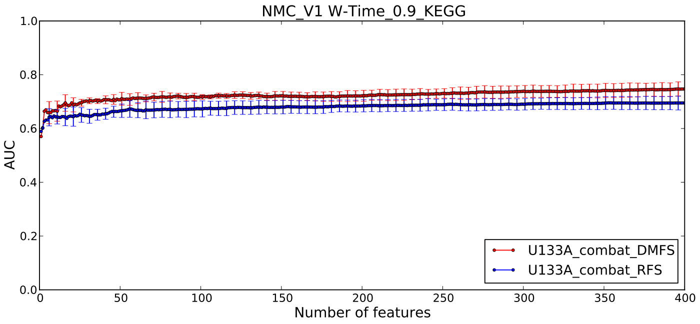

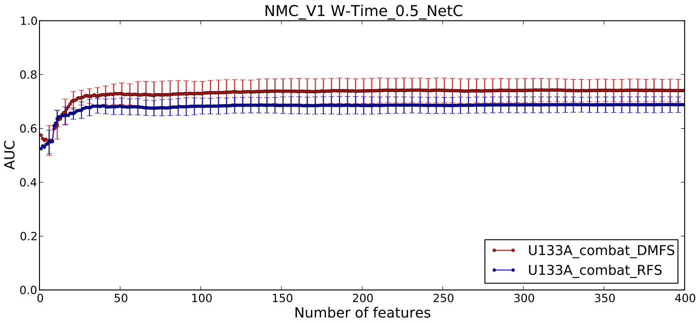

2.13 Winter-Time - HPRD9, damping factors 0.8 and 0.9

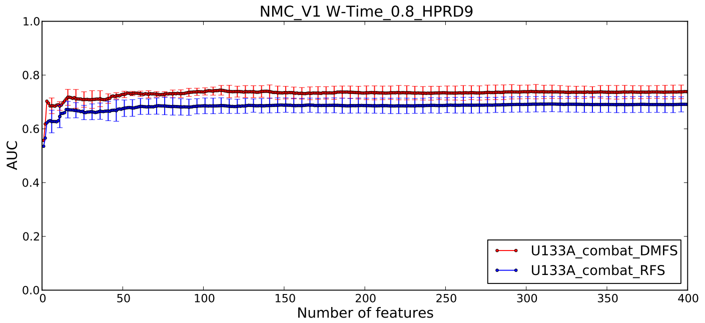

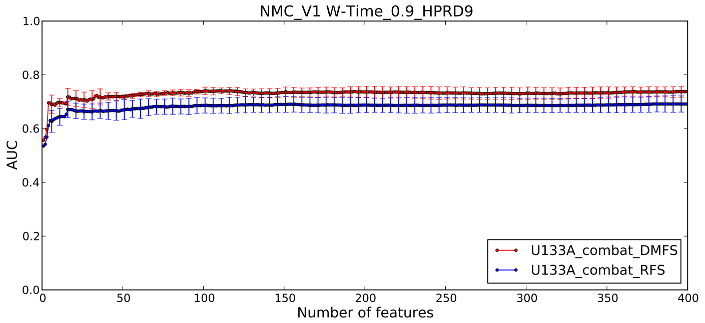

2.14 Winter-Time - I2D, damping factor 0.8

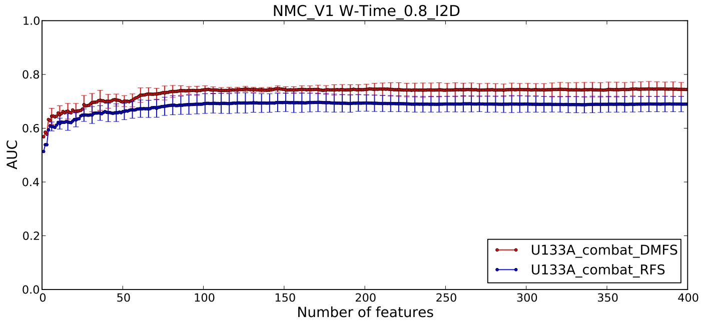

2.15 Winter - KEGG, damping factors 0.6 and 0.7

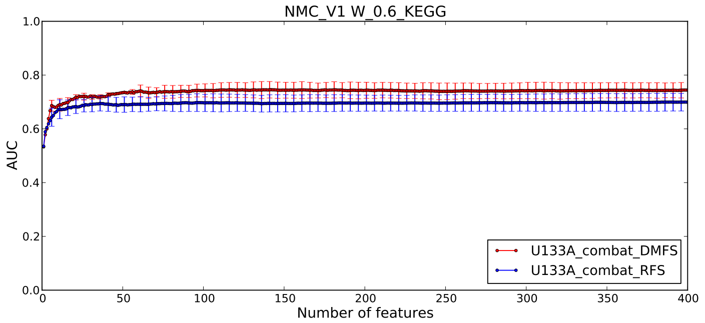

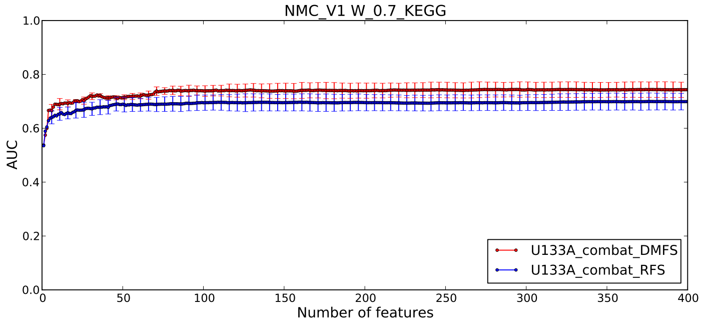

2.16 Winter - NetC, damping factors 0.1 and 0.8

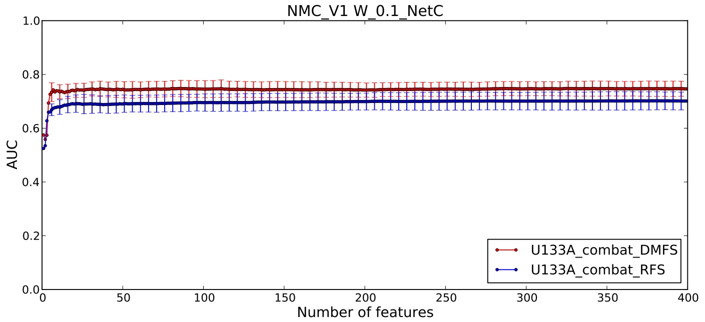

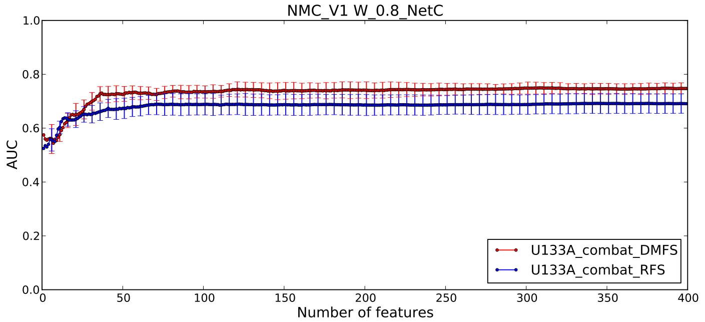

2.17 Winter - HPRD9, damping factors 0.4 and 0.5

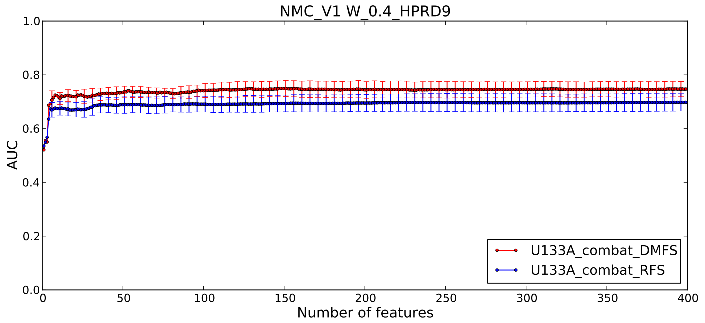

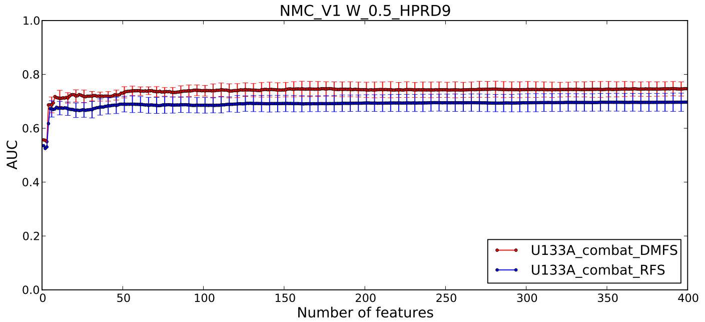

2.18 Winter - I2D, damping factor 0.8

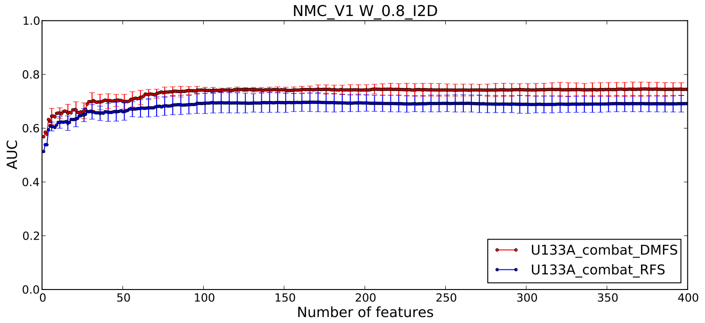

3 Winter and GeneRank - The damping factor does not have a major influence on the performance

The plots are similar to the plots above. We show the AUC value versus damping factor curves for the Winter method and GeneRank algorithm. Here we fixed the number of features to 50.

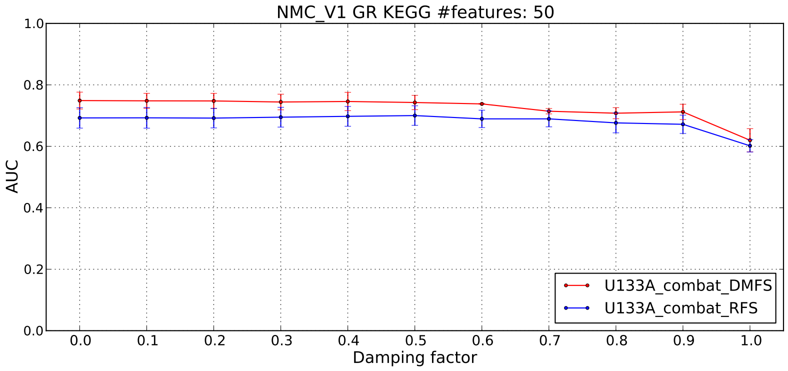

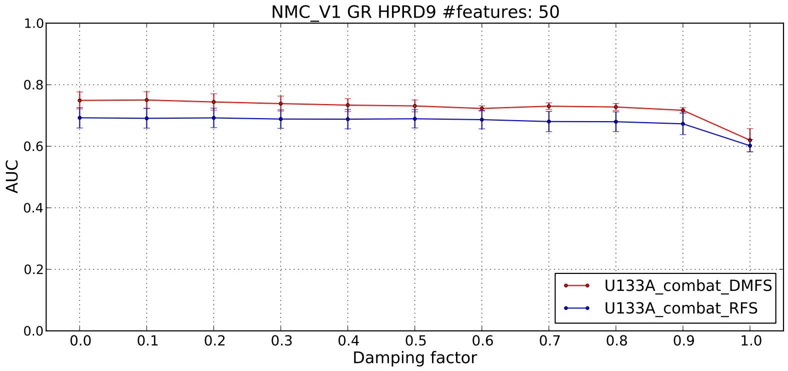

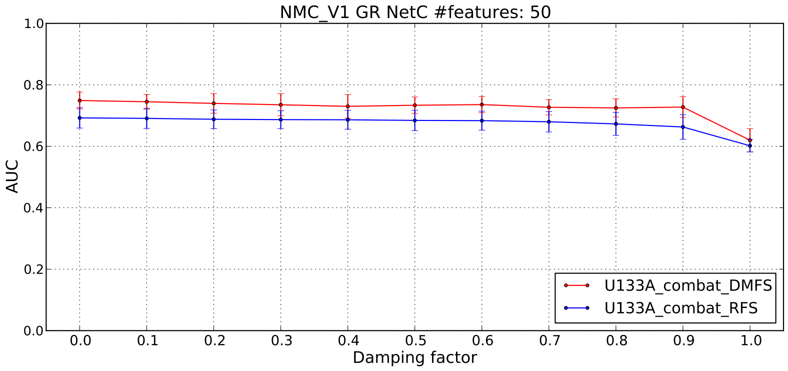

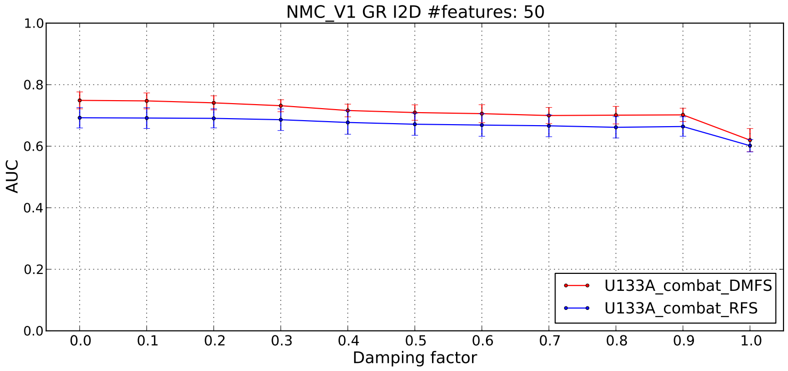

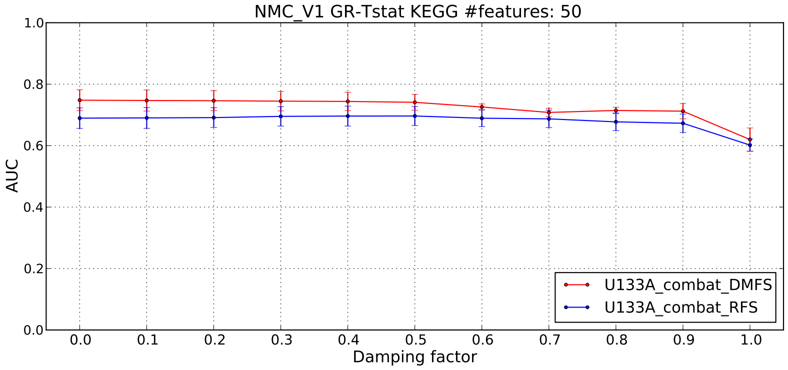
NMC_V1 GR-Tstat KEGG #features: 50
AUC
Damping factor
U133A_combat_DMFS
U133A_combat_RFS
0.0 0.1 0.2 0.3 0.4 0.5 0.6 0.7 0.8 0.9 1.0
0.0 0.2 0.4 0.6 0.8 1.0

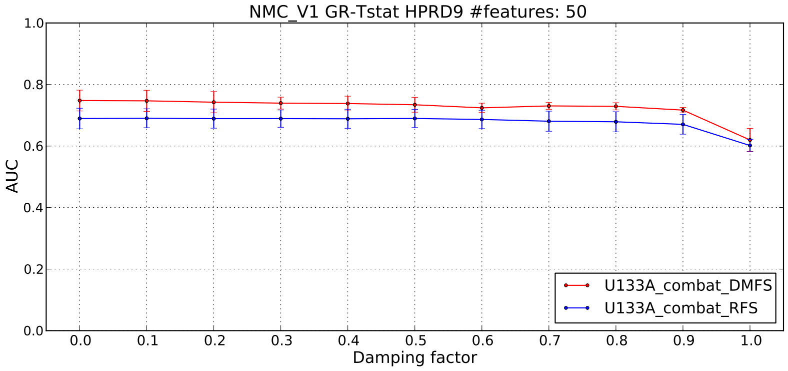
NMC_V1 GR-Tstat HPRD9 #features: 50
AUC
Damping factor
U133A_combat_DMFS
U133A_combat_RFS
0.0 0.1 0.2 0.3 0.4 0.5 0.6 0.7 0.8 0.9 1.0
0.0 0.2 0.4 0.6 0.8 1.0

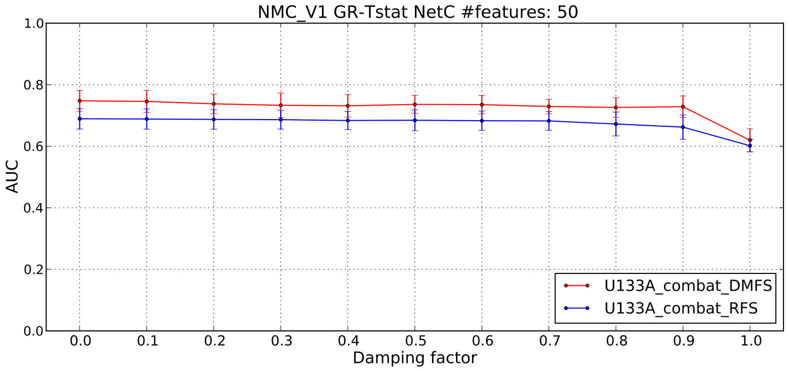
NMC_V1 GR-Tstat NetC #features: 50
AUC
Damping factor
U133A_combat_DMFS
U133A_combat_RFS
0.0 0.1 0.2 0.3 0.4 0.5 0.6 0.7 0.8 0.9 1.0
0.0 0.2 0.4 0.6 0.8 1.0

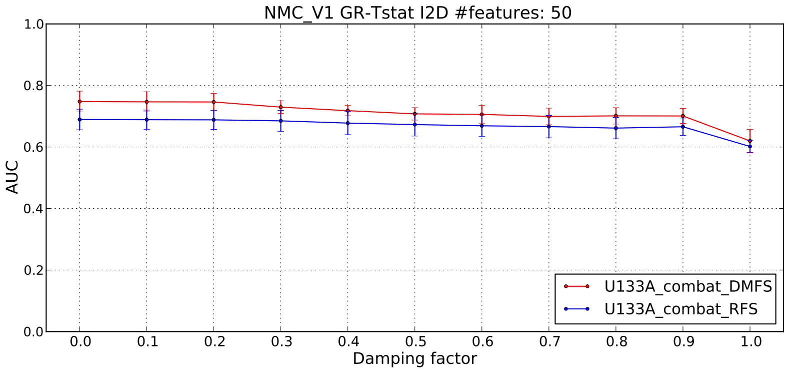
NMC_V1 GR-Tstat I2D #features: 50
AUC
Damping factor
U133A_combat_DMFS
U133A_combat_RFS
0.0 0.1 0.2 0.3 0.4 0.5 0.6 0.7 0.8 0.9 1.0
0.0 0.2 0.4 0.6 0.8 1.0

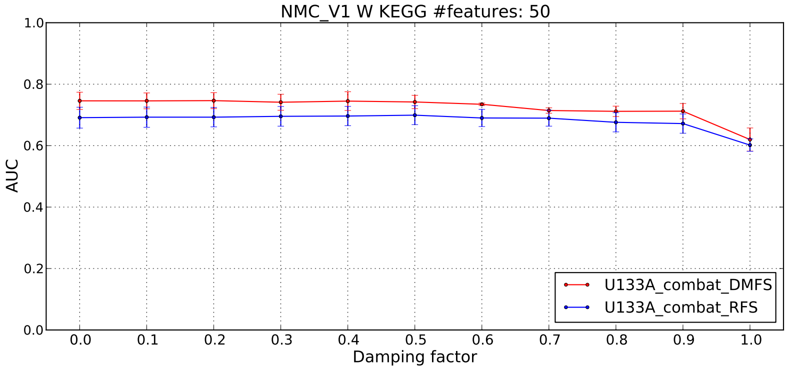
NMC_V1 W KEGG #features: 50
AUC
Damping factor
U133A_combat_DMFS
U133A_combat_RFS
0.0
0.2
0.4
0.6
0.8
1.0
0.0 0.1 0.2 0.3 0.4 0.5 0.6 0.7 0.8 0.9 1.0

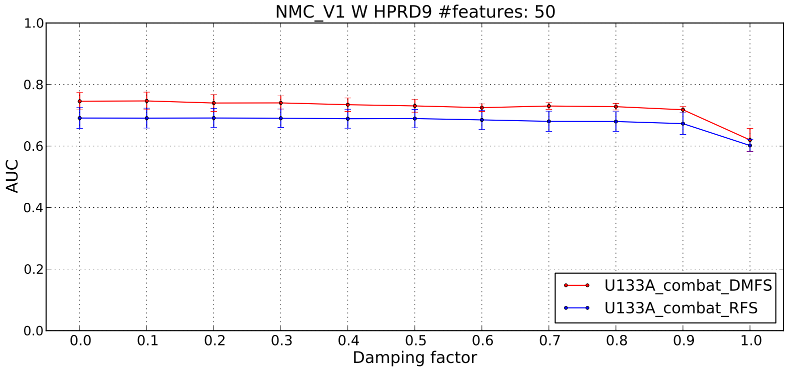
NMC_V1 W HPRD9 #features: 50
AUC
Damping factor
U133A_combat_DMFS
U133A_combat_RFS
0.0
0.2
0.4
0.6
0.8
1.0
0.0 0.1 0.2 0.3 0.4 0.5 0.6 0.7 0.8 0.9 1.0

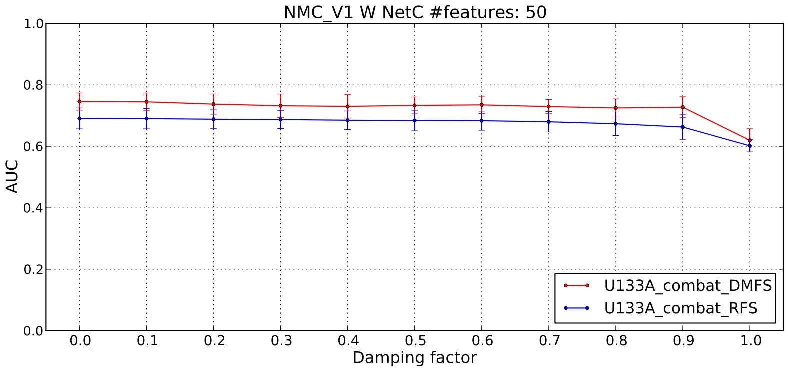
NMC_V1 W NetC #features: 50
AUC
Damping factor
U133A_combat_DMFS
U133A_combat_RFS
0.0
0.2
0.4
0.6
0.8
1.0
0.0 0.1 0.2 0.3 0.4 0.5 0.6 0.7 0.8 0.9 1.0

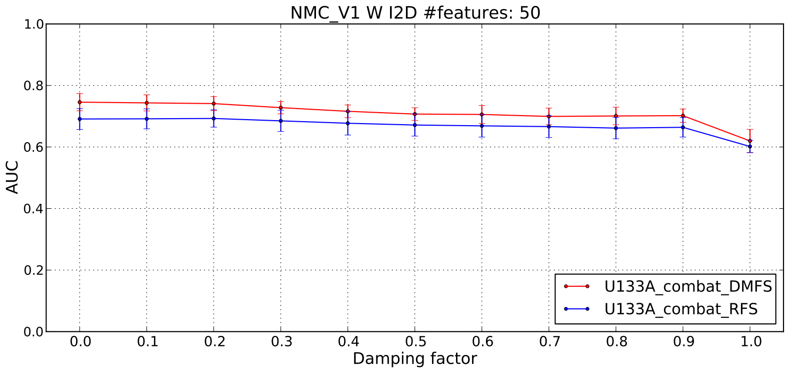
NMC_V1 W I2D #features: 50
AUC
Damping factor
U133A_combat_DMFS
U133A_combat_RFS
0.0
0.2
0.4
0.6
0.8
1.0
0.0 0.1 0.2 0.3 0.4 0.5 0.6 0.7 0.8 0.9 1.0

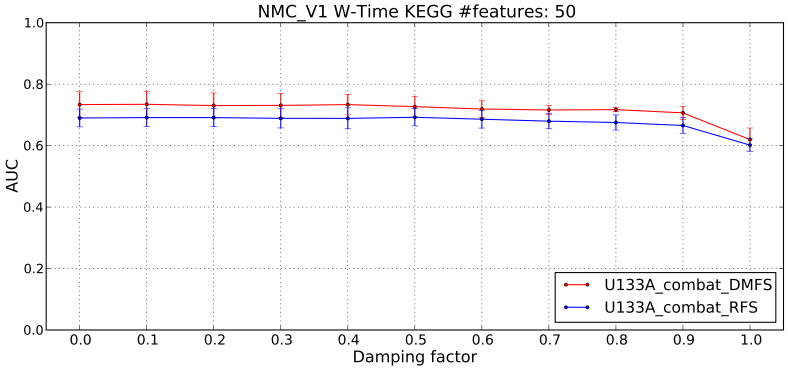
NMC_V1 W-Time KEGG #features: 50
AUC
Damping factor
U133A_combat_DMFS
U133A_combat_RFS

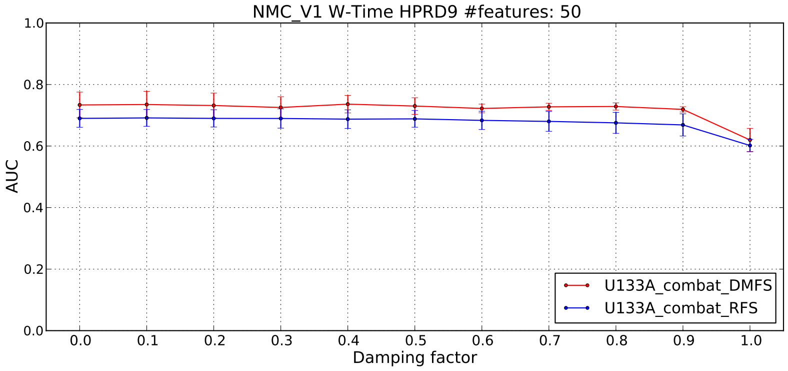
NMC_V1 W-Time HPRD9 #features: 50
AUC
Damping factor
U133A_combat_DMFS
U133A_combat_RFS

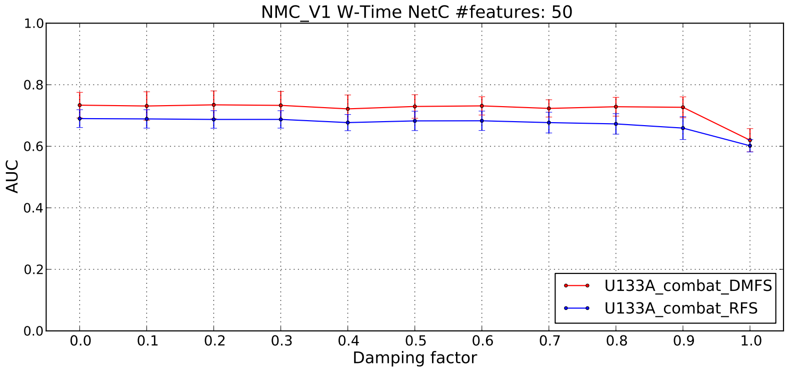
NMC_V1 W-Time NetC #features: 50
AUC
Damping factor
U133A_combat_DMFS
U133A_combat_RFS

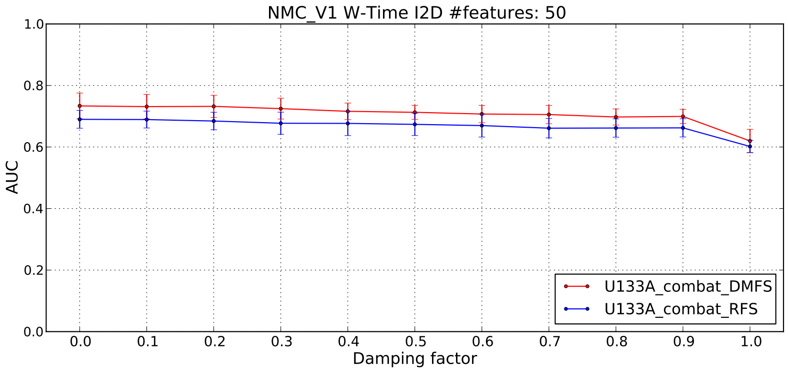
NMC_V1 W-Time I2D #features: 50
AUC
Damping factor
U133A_combat_DMFS
U133A_combat_RFS

4 Randomisation of the secondary data sources does not lead to worse classification performances

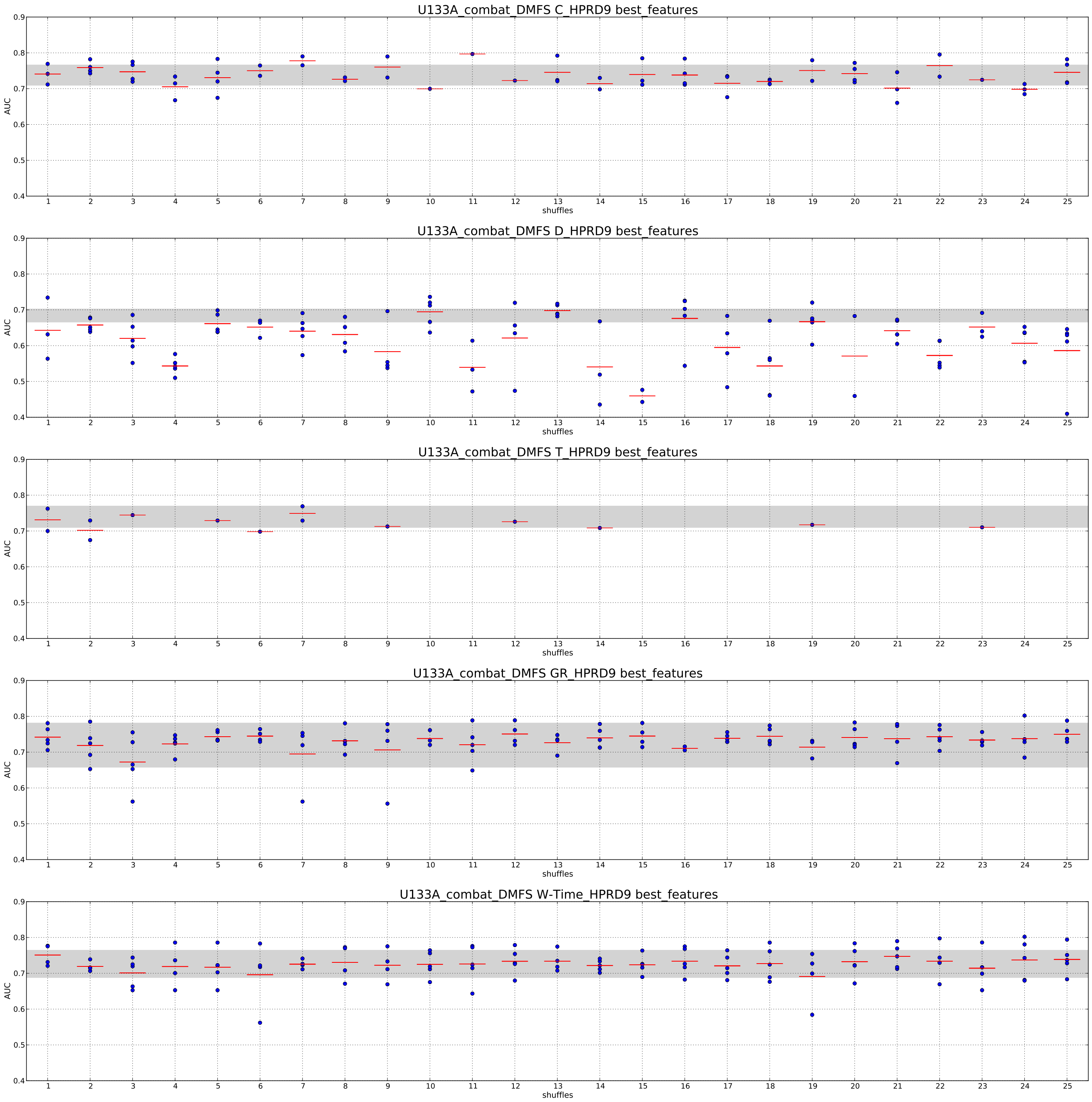

Figure 2: **Classification performance when employing randomized networks, HPRD9 and DMFS data**. The nodes in the HPRD9 PPI network were shuffled 25 times yielding 25 different randomized networks. Each network dependent feature selection method was applied to each of the randomized networks and classifiers were trained using the double-loop CV protocol. The grey area indicates the AUC interval employing the unshuffled PPI network. L - Lee, C - Chuang, T - Taylor, D - Dao, W - Winter using association between class labels and gene expression as initial gene ranks, W-time - Winter using association between survival times and gene expression as initial gene ranks, GR - GeneRank with absolute expression difference between 'good' and 'poor' outcome groups as initial rank, GR-Tstat - GeneRank employing the t-statistic as initial gene ranks.

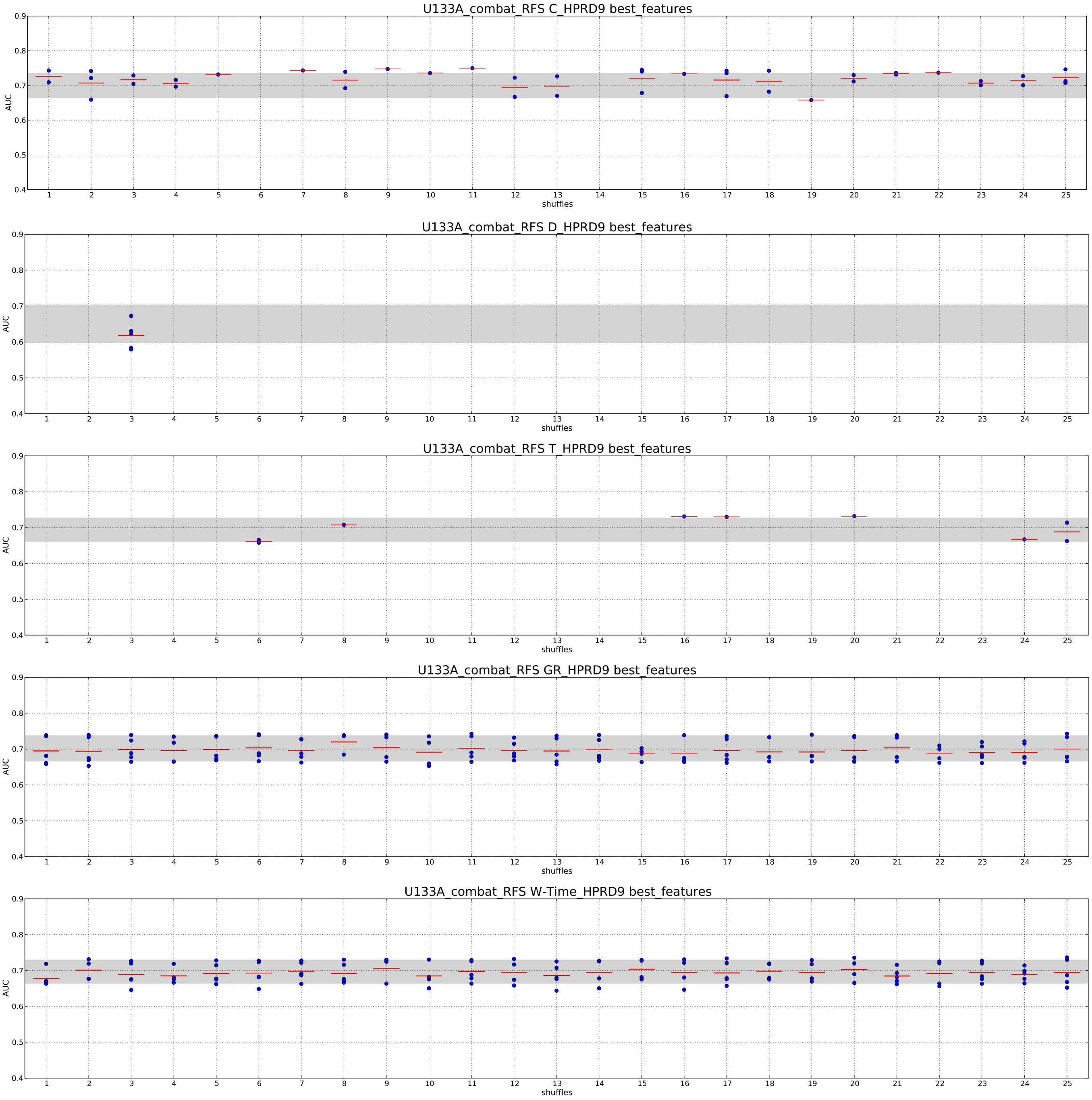

Figure 3: **Classification performance when employing randomized networks, HPRD9 and RFS data**. The nodes in the HPRD9 PPI network were shuffled 25 times yielding 25 different randomized networks. Each network dependent feature selection method was applied to each of the randomized networks and classifiers were trained using the double-loop CV protocol. The grey area indicates the AUC interval employing the unshuffled PPI network. L - Lee, C - Chuang, T - Taylor, D - Dao, W - Winter using association between class labels and gene expression as initial gene ranks, W-time - Winter using association between survival times and gene expression as initial gene ranks, GR - GeneRank with absolute expression difference between ‘good’ and ‘poor’ outcome groups as initial rank, GR-Tstat - GeneRank employing the t-statistic as initial gene ranks.

Table 2: **P-values of the Wilcoxon Rank test for the shuffled networks.** We compared the pooled AUC value distribution of all 25 randomized networks with the AUC value distribution of the unshuffled network.

Data set	Method	p-value	Data set	Method	p-value
RFS			DMFS		
	C_HPRD9	0.1164		C_HPRD9	0.7276
	C_I2D	0.8604		C_I2D	0.3798
	C_KEGG	0.4335		C_KEGG	0.8884
	C_NetC	0.7554		C_NetC	0.6313
	D_HPRD9	0.3095		D_HPRD9	0.0261
	D_I2D	0.0134		D_I2D	0.0126
	D_KEGG	0.0016		D_KEGG	0.0053
	D_NetC	0.0667		D_NetC	0.0122
	GR_HPRD9	0.8516		GR_HPRD9	0.8963
	GR_I2D	0.8848		GR_I2D	0.6129
	T_HPRD9	0.8981		T_HPRD9	0.3426
	T_I2D	0.1659		T_I2D	0.4464
	T_KEGG	0.8230		T_KEGG	0.2297
	T_NetC	0.1027		T_NetC	0.5941
	W-Time_HPRD9	0.6593		W-Time_HPRD9	0.5611
	W-Time_I2D	0.6765		W-Time_I2D	0.8972

4.0.1 Overlap of features determined on randomized networks

Table 3: **P-values of the Wilcoxon Rank test between the overlap of features determined on the randomized networks and the original distributions of the overlap.**

Data set	Method	p-value	Data set	Method	p-value
DMFS			RFS		
	Method	p-vals		Method	p-vals
	C_HPRD9	0.0001		C_HPRD9	0.1232
	C_I2D	0.0544		C_I2D	0.0176
	C_KEGG	0.2168		C_KEGG	0.0005
	C_NetC	0.0002		C_NetC	0.0687
	D_HPRD9	0.0000		D_HPRD9	0.0002
	D_I2D	0.0000		D_I2D	0.0000
	D_KEGG	0.0000		D_KEGG	0.0013
	D_NetC	0.0000		D_NetC	0.0000
	GR_HPRD9	0.7075		GR_HPRD9	0.7608
	GR_I2D	0.0135		GR_I2D	0.2100
	T_HPRD9	0.3706		T_HPRD9	0.0303
	T_I2D	0.6965		T_I2D	0.7509
	T_KEGG	0.0684		T_KEGG	0.1818
	T_NetC	0.7273		T_NetC	0.0062
	W-Time_HPRD9	0.0003		W-Time_HPRD9	0.0746
	W-Time_I2D	0.2297		W-Time_I2D	0.0003

5 Analysis of features generated with the real PPI networks and with the randomized PPI networks

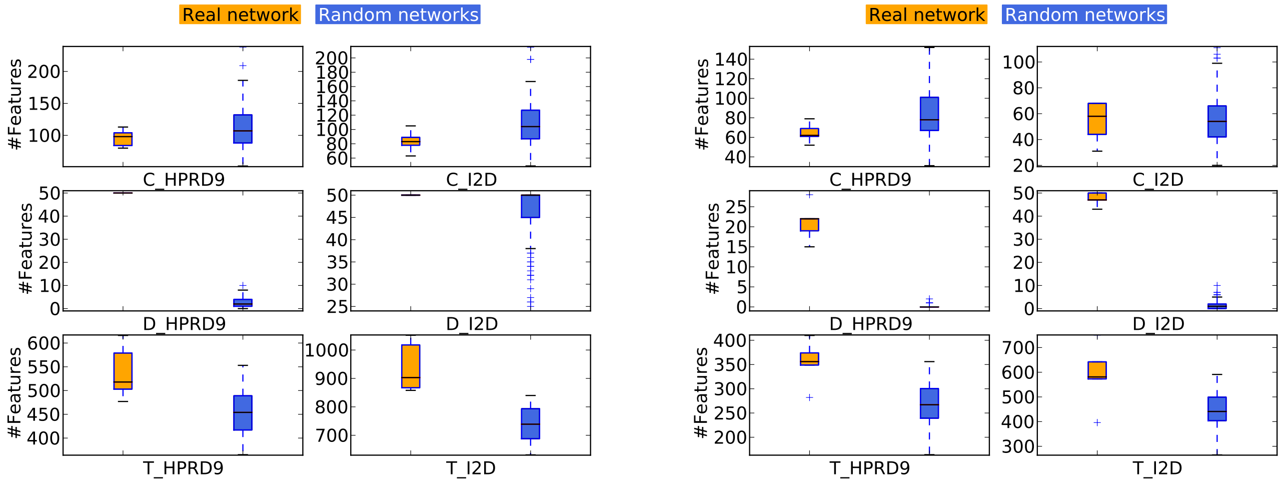

Figure 4: **The number of best features,** k. Classifiers were trained in the DLCV procedure yielding a different number of features k for each classifier. The figure shows a summary of all the ks for each method, once employing the real networks and once employing the 25 randomized networks. C - Chuang, T - Taylor, D - Dao. **Left:** DMFS, **right:** RFS.

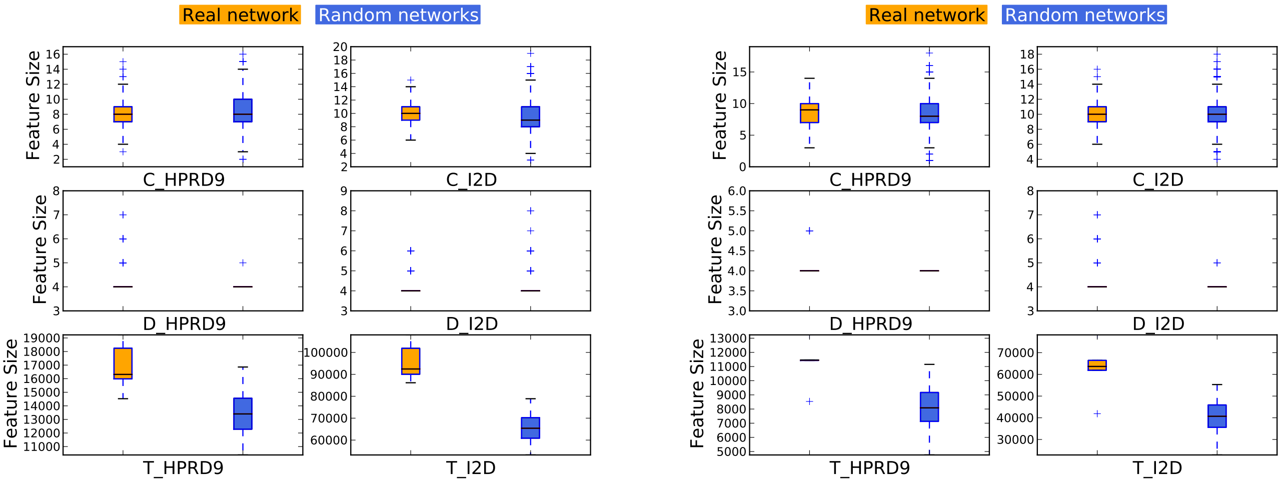

Figure 5: **The number of genes contained in the** k **best features**. C - Chuang, T - Taylor, D - Dao. **Left:** DMFS, **right:** RFS.

6 Tutorials

We give here the full example classes of the tutorial.

```
class NewFeatureExtractorFactory(object):

    productName = "NewFeatureExtractor"

    def __init__(self, yourParams):
        self.params = yourParams

    def train(self, dataset, network):
        return NewFeatureExtractor(dataset.geneLabels, features)
```

```
class NewFeatureExtractor(object):

    name                = "NewFeatureExtractor"
```

```python
    def __init__(self, geneLabels, features):

        #maps from geneLabels to features
        self.geneLabels          = geneLabels
        self.features            = features
        self.validFeatureCounts = range(1, len(self.features) + 1)

    # Score one feature for all patients
    @staticmethod
    def score(expressionData, feature):
        return score

    # Extract and score the features
    def extract(self, dataset, k):
        #Assert that the dataset is defined on the same genes as the feature extractor
        assert all(dataset.geneLabels == self.geneLabels)
        assert k in self.validFeatureCounts

        # Return the network scores for the k best composite features
        return numpy.transpose(numpy.array([self.score(dataset.expressionData, feature)
                for feature in self.features[:k]]))

    def toJsonExpression(self):
        #save the features and reload as json strings
        return json.dumps((self.__class__.__name__,
                [geneLabel for geneLabel in self.geneLabels],
                [sorted(feature) for feature in self.features]))
```

7 Fisher's exact test

To compare the overlap of gene sets extracted with the feature selection methods we employed Fisher's exact test. Here we illustrate how we applied this test. Given two sets of genes A and B drawn from a universe U we arrive at the following a contingency table:

	A	$\neg A$
B	$\|A \cap B\|$	$\|B \setminus A\|$
$\neg B$	$\|A \setminus B\|$	$\|U \setminus (A \cup B)\|$

(1)

To derive a p-value we first consider all 2×2 tables with the same marginal distribution. The p-value is then the relative frequency of all tables with a higher intersection $|A \cap B|$ than the tested table. Below we show an example why it is important to choose a test that takes the size of the universe into account.

Example 1 Given $|A| = 27$, $|B| = 35$ and the universe from which the gene sets were drawn has the size $|U| = 500$, assume the intersection $A \cap B$ contains 10 genes then

	A	$\neg A$
B	10	25
$\neg B$	17	458

is the contingency table for the Fisher's exact test with p-value $p = 2.318\text{E-}06$.
Now assume the same overlap was drawn from a much smaller universe–as it is the case when genes are restricted to the genes contained in a secondary data source–with $|U| = 100$. The corresponding contingency table

	A	$\neg A$
B	10	25
$\neg B$	17	58

would receive a p-value of $p = 0.3286$.

8 NMC metrics

The metric V2a considers the triangle consisting of the two class means and the sample. Given the Euclidian distance d the score is calculated as:

$$s(\text{sample}) = \frac{d(\mu_{\text{good}}, \text{sample})}{d(\mu_{\text{good}}, \text{sample}) + d(\text{sample}, \mu_{\text{poor}})} \tag{2}$$

where d is the Euclidian distance. The metric V2b is defined as:

$$s(\text{sample}) = \frac{d(\text{sample}, \mu_{\text{poor}}) - d(\text{sample}, \mu_{\text{good}})}{d(\mu_{\text{good}}, \mu_{\text{poor}})} \tag{3}$$

The fourth metric employs the cosine distance. The angle between the sample and the straight line from μ_{good} to μ_{poor} gives the score.

9 Randomising class labels

We performed two additional experiments to evaluate the influence of the class labels on the classification performance.

We employed the two expression data sets. One contains about 600 breast cancer samples labeled according to five-year distant metastasis free survival (DMFS, good outcome: 433 (69.50 %), poor outcome: 190 (30.50 %)); the other one contains about 1600 samples with the endpoint five-year recurrence free survival (RFS, good outcome: 1161 (71.84 %), poor outcome: 455 (28.16 %)). Note, that the DMFS data set is a subset of the RFS data set, i.e. samples and their gene expression are the same but the label, 'good' or 'poor' might vary.

We trained nearest-mean classifiers to assess the performance of features. Features were determined by the method 'Single Genes', where genes were ranked according to their absolute t-statistic between the two sample groups. The training and evaluation is described in Figure 1 of the main manuscript.

We executed two experiments: 'Shuffling class labels' and 'Randomising class labels'. In both cases the class labels of the outer CV's training data were altered. Subsequently, the number of best performing features k was trained in the inner CV employing incorrect class labels. The final classifier in the outer loop employed the top k ranking single genes. The ranking was also determined on the incorrectly labeled training data.

In this experiment the class labels were were redistributed randomly across the samples. By doing so the ratio between 'good' and 'poor' outcome patients stays the same as in the original data set. To add more statistical power we shuffled the class labels five times for each outer CV training data set. Thus we yield 25 AUC values and feature sets in the outer CV and execute the inner CV 25 times.

Given a percentage of 'poor' outcome samples, class labels–'good' and 'poor'–were created with respect to that ratio and randomly attached to the samples. Again, we created random class labels 25 times for each outer CV training data set.

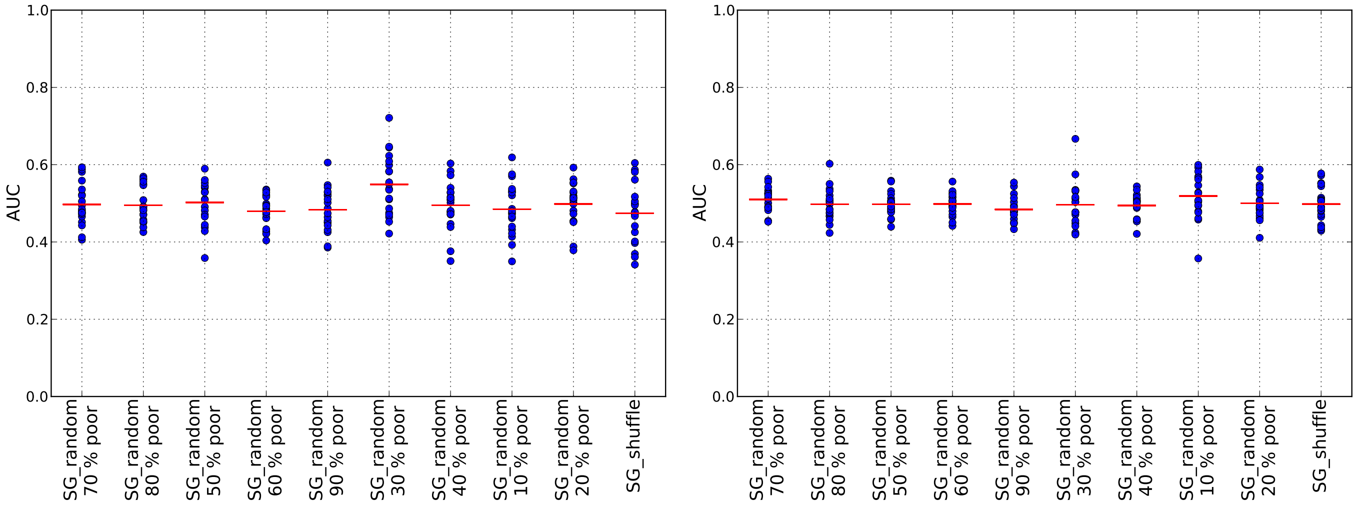

Figure 6: **Classification results obtained in the DLCV, employing a NMC and the data sets DMFS (left) and RFS (right).** The class labels of the outer loop testing data set have also been randomized/shuffled. AUC obtained when training single-genes classifiers with altered class labels.

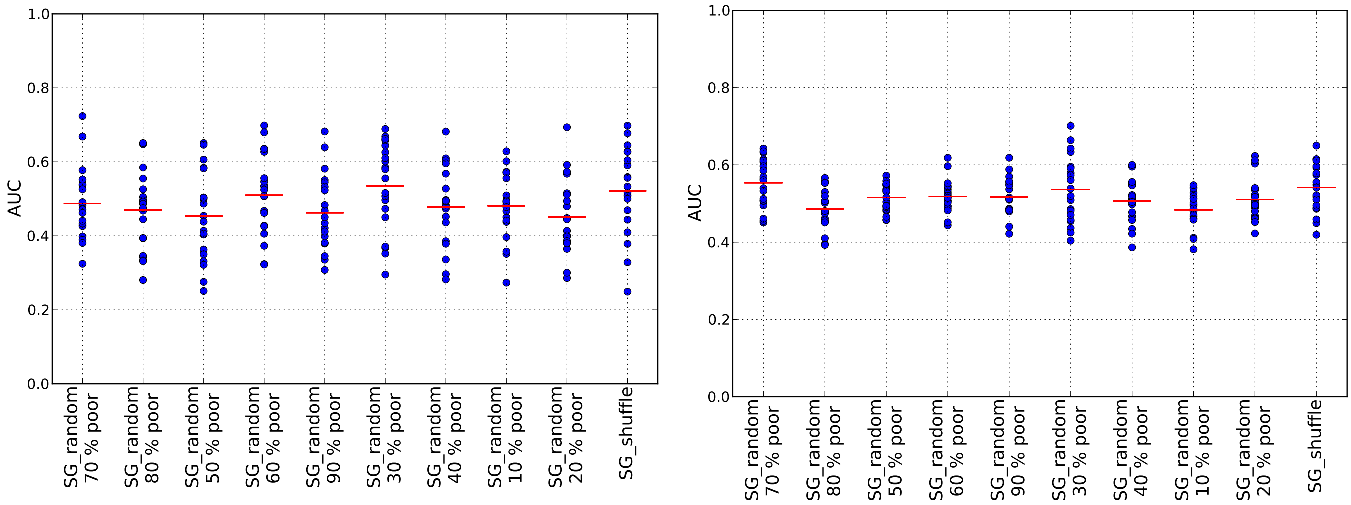

Figure 7: **Classification results obtained in the DLCV, employing a NMC and the data sets DMFS (left) and RFS (right).** The original class labels for the outer loop testing data set have been employed.

Figure 6 shows the classification result when classifiers were trained and tested on data, that carries randomized class labels, whereas Figure 7 shows the classification results when classifiers were trained on data carrying randomized class labels but were tested with correctly labeled data.

- The average AUC value is as expected 0.5. However, some random labels give a better performance than expected, e.g. when 30% of the patients are forced to carry a poor outcome label on the DMFS data set.
- All random label combinations are different from the real labeling. However, since the data sets are heavily imbalanced with respect to the patient outcome many of the good outcome patients are labeled correctly, which in turn might lead to a better performance than random (AUC of 0.5%).
- When 70% of the patients carry a poor outcome label, the real relation between poor and good outcome patients is reversed. Still, the AUC distribution looks similar to all the other distributions.
- Figure 6: the classifiers perform better when the ratio between good and poor outcome patients is similar to the real proportions. In all other cases the proportion between good and poor patients does not play a role, which is unexpected. This might be due to only repeating the shuffling/randomisation five times.
